# Targeting transferrin receptors at the blood-brain barrier improves the uptake of immunoliposomes and subsequent cargo transport into the brain parenchyma

**DOI:** 10.1038/s41598-017-11220-1

**Published:** 2017-09-04

**Authors:** Kasper Bendix Johnsen, Annette Burkhart, Fredrik Melander, Paul Joseph Kempen, Jonas Bruun Vejlebo, Piotr Siupka, Morten Schallburg Nielsen, Thomas Lars Andresen, Torben Moos

**Affiliations:** 1 Laboratory for Neurobiology, Biomedicine, Institute of Health Science and Technology, Aalborg University, Aalborg, Denmark; 2 Center for Nanomedicine and Theranostics, Department of Micro- and Nanotechnology, Technical University of Denmark, Lyngby, Denmark; 3 Department of Biomedicine, Aarhus University, Aarhus, Denmark

## Abstract

Drug delivery to the brain is hampered by the presence of the blood-brain barrier, which excludes most molecules from freely diffusing into the brain, and tightly regulates the active transport mechanisms that ensure sufficient delivery of nutrients to the brain parenchyma. Harnessing the possibility of delivering neuroactive drugs by way of receptors already present on the brain endothelium has been of interest for many years. The transferrin receptor is of special interest since its expression is limited to the endothelium of the brain as opposed to peripheral endothelium. Here, we investigate the possibility of delivering immunoliposomes and their encapsulated cargo to the brain via targeting of the transferrin receptor. We find that transferrin receptor-targeting increases the association between the immunoliposomes and primary endothelial cells *in vitro*, but that this does not correlate with increased cargo transcytosis. Furthermore, we show that the transferrin receptor-targeted immunoliposomes accumulate along the microvessels of the brains of rats, but find no evidence for transcytosis of the immunoliposome. Conversely, the increased accumulation correlated both with increased cargo uptake in the brain endothelium and subsequent cargo transport into the brain. These findings suggest that transferrin receptor-targeting is a relevant strategy of increasing drug exposure to the brain.

## Introduction

The fragile brain parenchyma is protected from the periphery by the blood-brain barrier (BBB), which consists of endothelial cells that interconnect tightly through tight junction proteins^1^. Therefore, transport of molecules into the brain parenchyma is severely restricted^2^. Due to the BBB, drug delivery to the brain sustains a major challenge for effective treatment of brain diseases despite development of numerous drug compounds and nanomedicines that putatively fit this purpose^3^. Many of these approaches base their brain accumulation strategies on targeting of the transferrin receptor^4^. The transferrin receptor is responsible for the transport of iron into the brain parenchyma to maintain iron homeostasis that is of great importance for metabolism, neural conductivity, and hence, proper brain function^5^. The transferrin receptor is an interesting and unique target, since it is exclusively expressed on the endothelial cells of the brain capillaries and not on endothelial cells lining the vessels in other tissues^6^. Evidence presented in the recent years suggests that the process of iron transport through the BBB does not include full transport of the transferrin receptor to the abluminal side, which instead only seems to become internalized and either transported back to the luminal membrane, or sorted to the intracellular stores of residing transferrin receptors. From here, they can quickly become mobilized to provide adequate iron transport in conditions where increased amounts are needed^7–10^. Full transcytosis of nanomedicines via this receptor route thus seems unlikely^11^. Still, overwhelming amounts of preclinical evidence exist to suggest that various types of drug constructs or nanomedicines can improve several disease conditions via targeting of the transferrin receptor, hence, illustrating a discrepancy between these two fields of research^12^.

We previously showed that targeting the transferrin receptor using a high affinity antibody leads to great association between the antibody and the transferrin receptors of the brain capillaries *in vivo*, but also that the antibodies were retained in the vascular compartment after brain capillary depletion^13^. The same observations hold true when targeting the transferrin receptor with antibody-conjugated liposomes^14^. In the present study, we have taken a parallel approach by investigating either fluorescently labelled or oxaliplatin-loaded transferrin receptor-targeted immunoliposomes for their association to, and transport across, the BBB *in vitro* and *in vivo*. We quantify the association between immunoliposomes and brain capillary endothelial cells (BCECs) and their transport across these cells using flow cytometry and platinum content quantification in a primary rat *in vitro* model of the BBB. Furthermore, we investigate the uptake of fluorescently labelled immunoliposomes both *in vitro* and *in vivo* using spinning disk confocal microscopy. Lastly, we study the potential of transferrin receptor-targeting to increase the transport of a liposome-encapsulated cargo (oxaliplatin) into the brain parenchyma after intravenous injection into young rats, and provide quantitative data for brain uptake after capillary depletion in combination with circulation time profiling and biodistribution analysis.

## Results

### Primary brain capillary endothelial cells express blood-brain barrier characteristics and transferrin receptors *in vitro*

**Figure 1:**
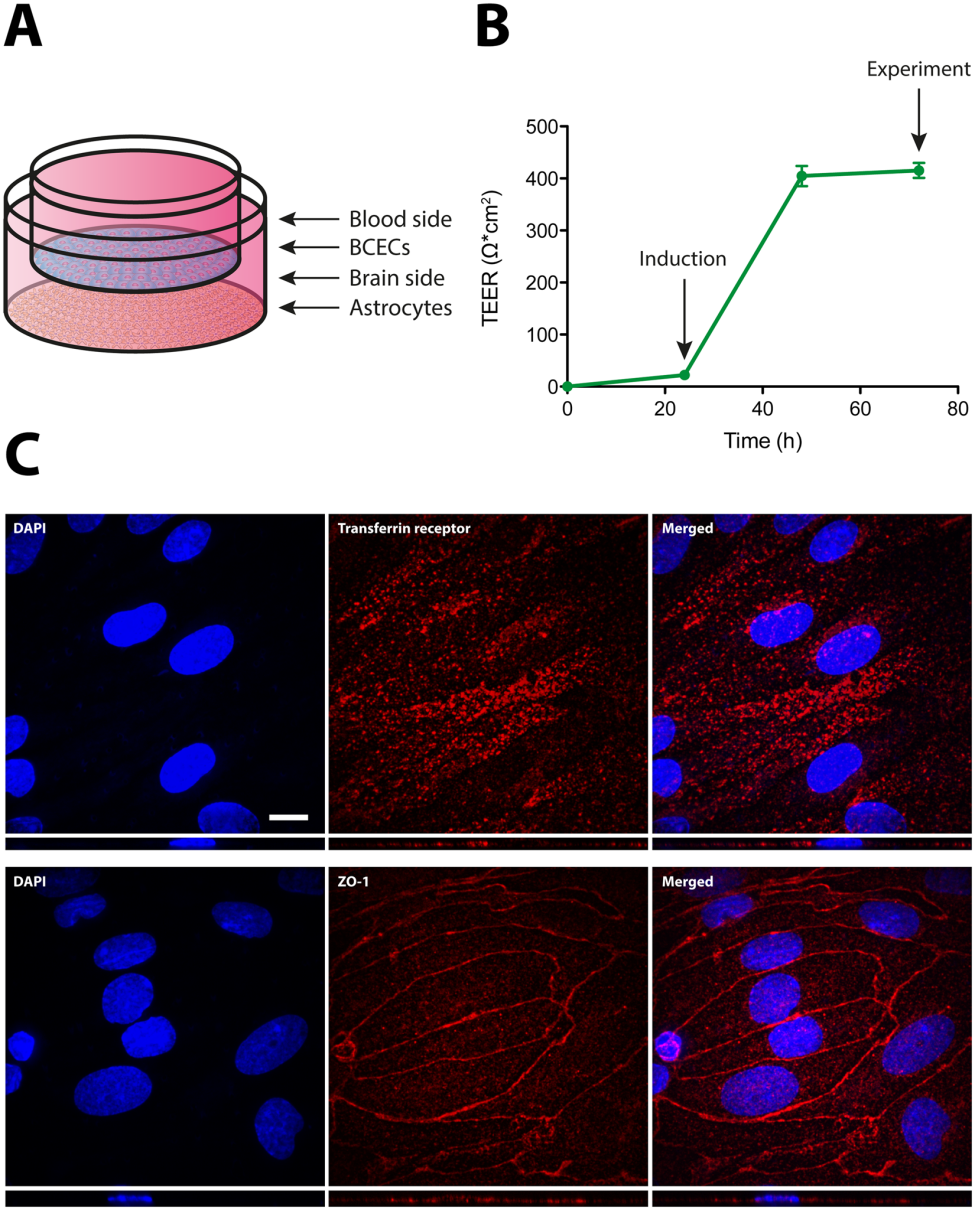
Setup and characterization of the *in vitro* model of the BBB based on primary rat BCECs and astrocytes. (**A**) Primary BCECs derived from young rats were setup in Transwell co-culture with astrocytes, hereby yielding a polarized layer of BCECs to be used for uptake and transcytosis experiments. (**B**) TEER was measured continuously to evaluate the tightness of the *in vitro* BBB model. The TEER values were approximately 400 Ω*cm^2^ at the time of the experiments, and this value was stabile throughout the duration of any experiment. (**C**) After reaching high TEER values, the resulting tight BCEC monolayers expressed both transferrin receptors (upper panel) and the TJ-related protein, ZO-1 (lower panel). The positive staining for the transferrin receptors was found associated with the luminal membrane as well as in the cell cytoplasm, whereas the ZO-1 staining presented as a homogenous lining of the intercellular junctions, suggesting the presence of functional tight junctions. Scale bar depicts 10 µm. BBB: Blood-brain barrier. BCEC: Brain capillary endothelial cells. TEER: Transendothelial electrical resistance. DAPI: Diamino-phenylindole. TJ: Tight junction. ZO-1: Zonula occludens 1.

To study the interaction between transferrin receptor-targeted (OX26) immunoliposomes and BCECs *in vitro*, we employed a primary rat *in vitro* model of the BBB, which consisted of a co-culture between BCECs and astrocytes (Fig. 1A). BBB characteristics were induced as described previously^15^, and the transendothelial electrical resistance (TEER) was measured to evaluate the tightness of the resulting barrier. TEER values were on average 400 Ω*cm^2^ when initiating the experiments (Fig. 1B), and no reduction in TEER could be measured after incubation with the different immunoliposomal formulations (Supplementary Fig. S5). The BCECs expressed both transferrin receptors (Fig. 1C, upper panel) and the TJ-related protein, ZO-1 (Fig. 1C, lower panel). The positive staining for the transferrin receptors was found to be associated with the luminal membrane as well as in the cell cytoplasm, which are the known locations of the transferrin receptors in BCECs *in vivo* (Fig. 1C, upper panel). The ZO-1 staining presented as a clearly defined lining of the intercellular junctions, which in combination with the high TEER value is indicative of functional tight junctions in the model (Fig. 1C, lower panel).

### Transferrin receptor-targeting increases uptake of immunoliposomes in brain capillary endothelial cells *in vitro*

**Figure 2:**
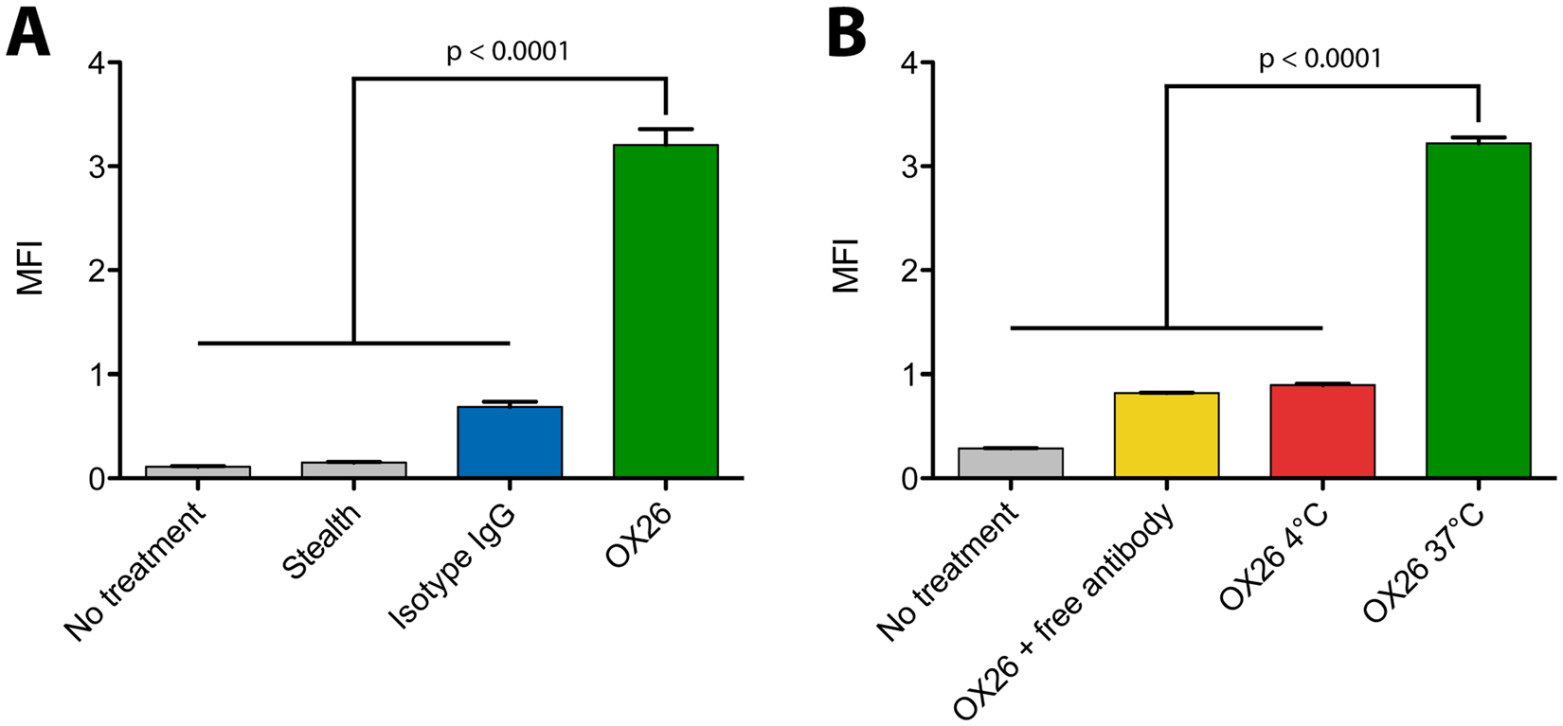
Flow cytometry evaluation of the association between immunoliposomes and BCECs *in vitro*. (**A**) BCECs were incubated with either stealth liposomes, isotype IgG, or OX26 immunoliposomes labelled with a fluorophore in the lipid membrane, and the treated cells were analyzed by flow cytometry to evaluate the association. OX26 immunoliposomes had a five-fold higher association compared to the isotype IgG immunoliposomes, whereas stealth liposomes had no association above the background of untreated cells (p < 0.0001). Due to the large difference in association between the two control liposomes, isotype IgG immunoliposomes was chosen as the most relevant control for subsequent experiments. (**B**) The association between BCECs and OX26 immunoliposomes was further characterized by co-incubation with free OX26 antibodies, which decreased the association significantly. Furthermore, incubation at 4°C also reduced the association, indicating an energy-demanding uptake mechanism (p < 0.0001). Data are presented as mean + SEM (n = 4–8), and the p-values depicted were derived from a one-way ANOVA with Tukey’s multiple comparisons post hoc test. MFI: Median fluorescence intensity.

**Figure 3:**
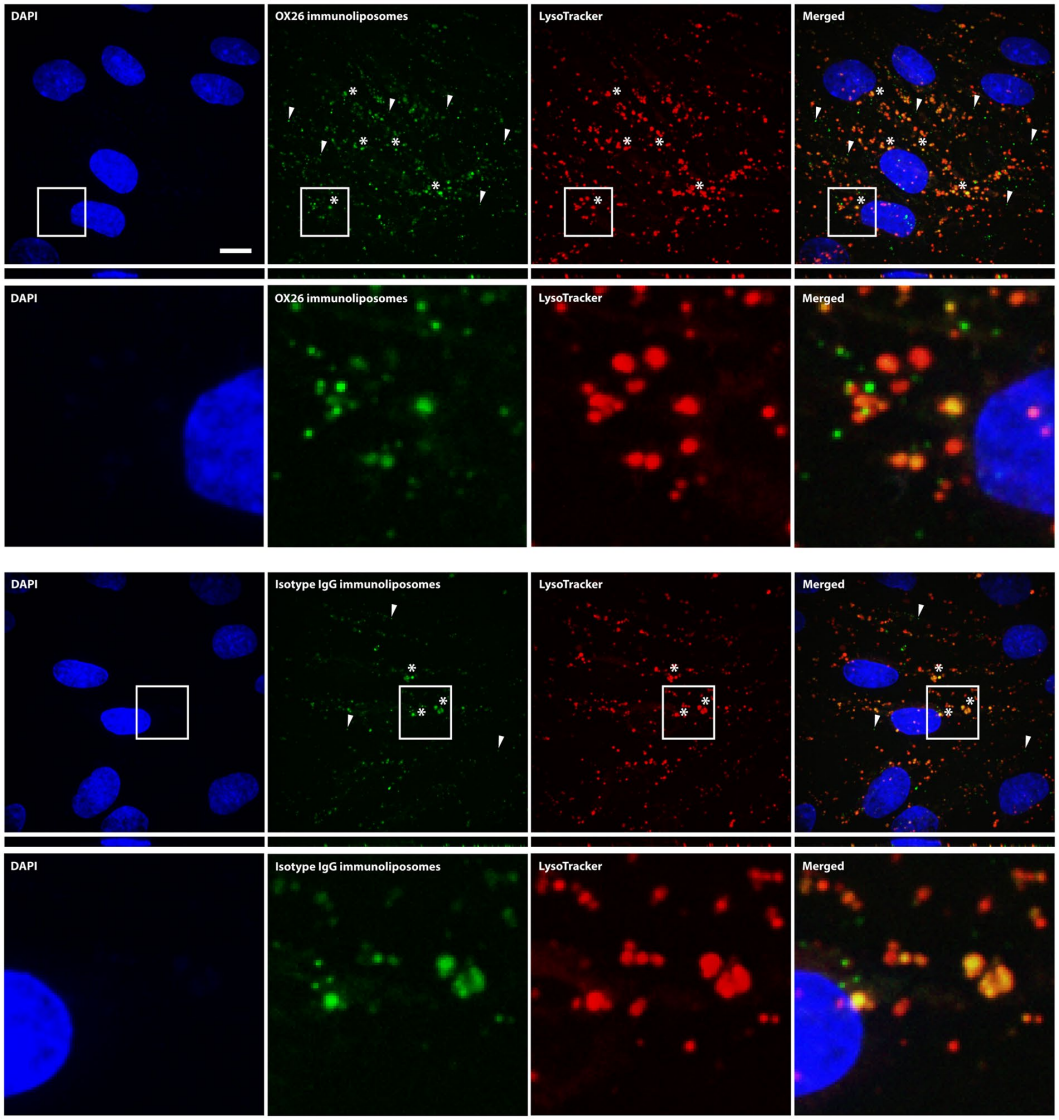
Spinning disk confocal microscopy images of immunoliposome-treated, primary rat BCECs. Treatment with either OX26 (upper panel) or isotype IgG (lower panel) immunoliposome revealed a clear association between the fluorescently labelled immunoliposomes and the BCECs. Morphologically, the fluorescent signals were either particulate in the periphery of the cells (arrows) or clustered into larger structures in the perinuclear area (asterisks). Counterstaining with LysoTracker revealed that these larger structures were lysosomes that the endocytosed immunoliposomes had been sorted to. The smaller, particulate signal (arrows) did not co-localize with the lysosomes, suggesting these to be newly endocytosed immunoliposomes. Scale bar depicts 10 µm. DAPI: Diamino-phenylindole.

After preparation, the immunoliposomes had an average hydrodynamic diameter of approximately 140 nm and a negative surface charge. The average number of antibodies per liposome was approximately 50 (Table 1). Furthermore, only minor leakage of oxaliplatin was observed during the post-insertion process and upon storage, and morphological assessment of the immunoliposomes revealed that they were unilamellar and spheroid-shaped (Supplementary Fig. S6). After incubation with OX26 immunoliposomes and control (isotype IgG and stealth) liposomes, the association between the different liposome formulations and the BCECs was analysed by flow cytometry. As depicted in Fig. 2A, OX26 immunoliposomes associated strongly with the BCECs, which corresponded to a five-fold increase in median fluorescent intensity compared to isotype IgG immunoliposomes (p < 0.0001). Stealth liposomes associated only weakly compared to the non-treated cells. The association between OX26 immunoliposomes and the BCECs could be out-competed with co-incubation with free OX26 antibodies, and likewise, incubation at 4°C reduced the median fluorescent intensity of the cells (Fig. 2B, p < 0.0001). This suggested that a large part of the OX26 immunoliposomes had been internalized via a saturable receptor-mediated mechanism during the 2 hours of incubation. Given the much higher avidity of the immunoliposomes compared to free antibodies, the ability of the free antibodies to out-compete the uptake was likely due to a difference in the diffusion properties and a difference in the impact of the diffusion barrier depicted by the glycocalyx of the BCECs. The great interaction between OX26 immunoliposomes and the BCECs could also be visualized by spinning disk confocal microscopy, where Z-stack projections revealed fluorescent signal from the immunoliposomes in the intracellular compartment of the BCECs (Fig. 3, upper panel). Smaller particulate signals were detected in the perimeter of the BCECs (arrows), whereas larger clusters of fluorescent signal (asterisks) were detected in the perinuclear region (Fig. 3, upper panel). By co-incubating with LysoTracker, we observed a high degree of co-localization between the OX26 immunoliposomes and lysosomes in the perinuclear region (asterisks), as well as isolated immunoliposome signals (arrows) in the periphery of the BCECs (Fig. 3, upper panel). The same observations were seen in BCECs treated with isotype IgG immunoliposomes (Fig. 3, lower panel). Counterstaining against the early endosome marker, EEA-1, revealed some co-localization, but these data were clearly affected by dissolution of the liposome membrane during immunocytochemistry processing (Supplementary Fig. S7).

**Table 1:** Characteristics of the oxaliplatin-loaded immunoliposomes. DLS: Dynamic light scattering. PDI: Polydispersity index. OxPt: Oxaliplatin.

Sample	Size (determined by DLS)	PDI	Zeta potential	Antibodies/liposome	OxPt concentration
OX26	139.3 ± 1.5 nm	0.127 ± 0.017	−21.89 ± 1.03 mV	47 ± 9	1.27 mg/mL
Isotype IgG	139.8 ± 2.0 nm	0.111 ± 0.023	−22.79 ± 1.58 mV	52 ± 7	1.27 mg/mL

### Transferrin receptor-targeting increases the uptake of an encapsulated cargo into brain capillary endothelial cells, but does not improve transendothelial transport *in vitro*

**Figure 4:**
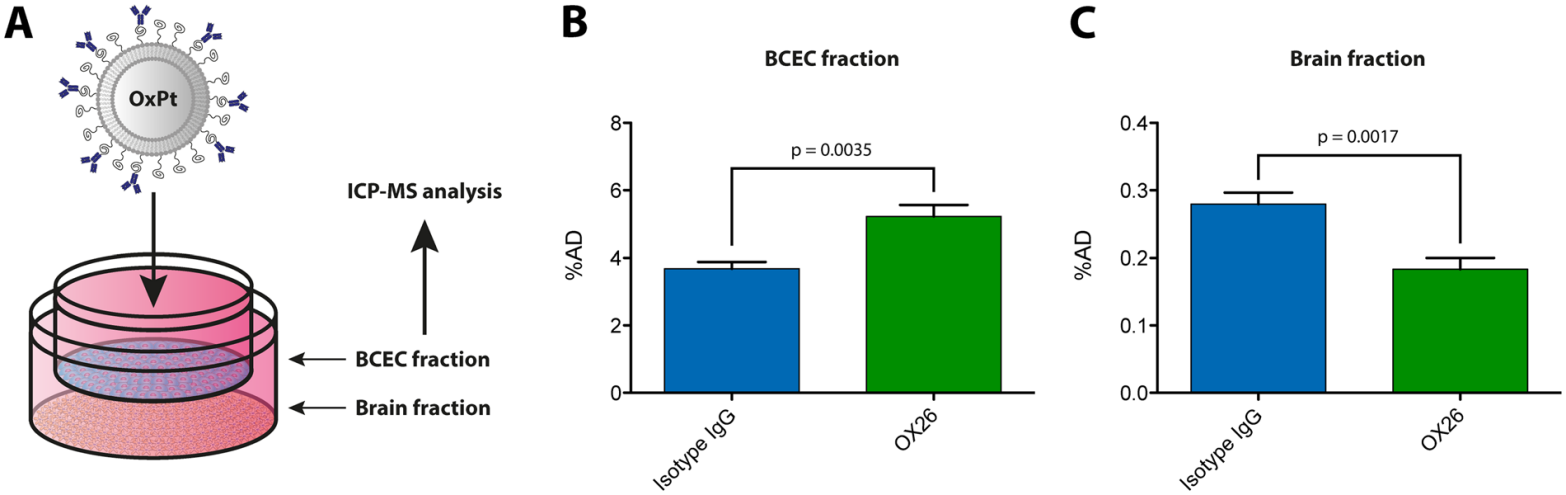
Transcytosis of oxaliplatin-loaded immunoliposomes across an *in vitro* model of the BBB. (**A**) Oxaliplatin-loaded immunoliposomes functionalized with either OX26 or isotype IgG antibodies were administrated to a monolayer of primary BCECs derived from young rats in Transwell co-culture with astrocytes. After incubation, the ‘BCEC’ and ‘brain’ fractions were isolated for subsequent ICP-MS analysis to quantify the platinum content. (**B**) In the BCEC fraction, OX26 immunoliposomes yielded a higher platinum content compared to the isotype IgG immunoliposomes (p = 0.0035). (**C**) In the brain fraction, the platinum content of the isotype IgG immunoliposome-treated group was significantly higher compared to that treated with OX26 immunoliposomes (p = 0.0017). Data are presented as mean + SEM (n = 7–8), and the p-values depicted were derived from a one-way ANOVA with Tukey’s multiple comparisons post hoc test. BCEC: Brain capillary endothelial cell. ICP-MS: Inductively-coupled plasma mass spectrometry. OxPt: Oxaliplatin.

To study the uptake of the immunoliposomes in greater detail, we antibody-functionalized oxaliplatin-loaded liposomes and administrated these to the primary rat *in vitro* model of the BBB. After 4 hours of incubation, the ‘BCEC’ and ‘brain’ fractions (cell culture medium of the bottom well) were collected and analyzed for their platinum content, and compared to the initial concentration in the Transwell insert (Fig. 4A). Targeting the transferrin receptor with OX26 immunoliposomes led to a higher platinum content in the BCECs compared to isotype IgG immunoliposomes, hereby underscoring the findings in the flow cytometry analysis (Fig. 4B, p = 0.0035). However, the magnitude of the difference was much lower compared to the observations from the flow cytometry experiments (Fig. 2A). When measuring the amount of platinum that had transcytosed through the BCEC layer during the incubation, we consistently found that the isotype IgG immunoliposomes delivered a higher amount compared to the OX26 immunoliposomes (Fig. 4C, p = 0.0017), which may be attributed to the static conditions of these experiments, or the long incubation time, chosen to confidently measure the platinum concentrations in the ‘brain’ fractions (the media samples from the bottom chamber of the Transwell co-culture setup). This did, however, not affect the TEER of the BCEC layer (Supplementary Fig. S5).

### Transferrin receptor-targeting increases the association between immunoliposomes and brain capillaries *in vivo* without evidence of liposome or ligand transcytosis

**Figure 5:**
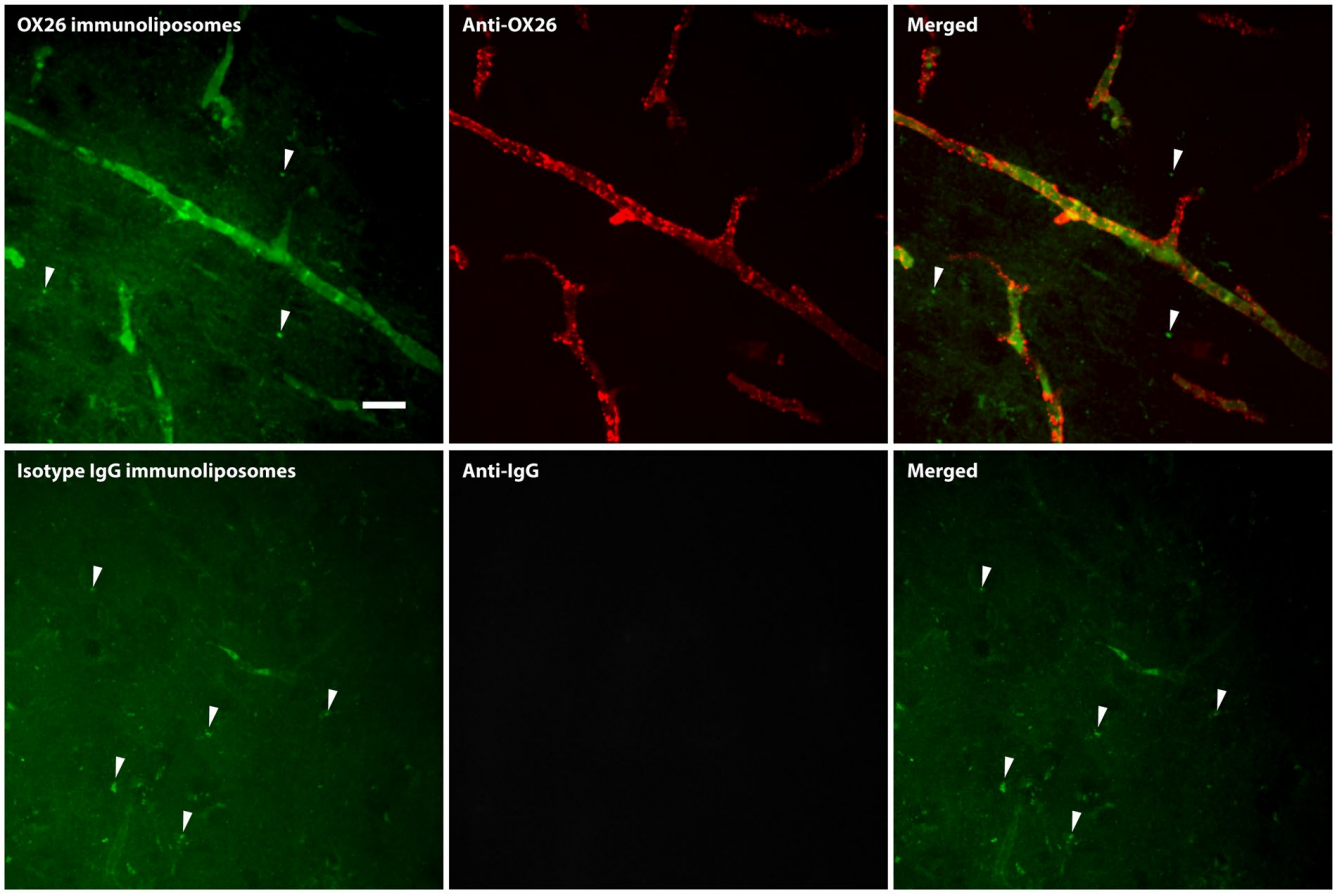
Uptake of fluorescently labelled immunoliposomes in brain capillaries *in vivo* evaluated by spinning disk confocal microscopy. Young rats were injected with either OX26 (upper panel) or isotype IgG (lower panel) immunoliposomes to assess their potential of interacting with the capillaries of the brain. OX26 immunoliposomes (upper panel) showed a good association to the microvessel structures of the brain, although the fluorescent intensity of the immunoliposomes were very low. Counterstaining against the OX26 antibody revealed that the immunoliposome and ligand had accumulated in the brain capillaries, but no sign of transcytosis could be detected. Isotype IgG immunoliposomes (lower panel) had almost no association to the microvessel structures of the brain, which was also evident from the counterstaining against mouse IgG, which did not present with the same vessel pattern as the OX26 immunoliposomes. In both cases, artefacts from the paraformaldehyde fixation could easily be detected (arrows), which was not regarded as a transcytosed immunoliposome, since the same was observable in non-treated animals (data not shown). Scale bar depicts 20 µm.

To study the targeting abilities of the OX26 immunoliposomes *in vivo*, we intravenously injected fluorescently labelled immunoliposomes and allowed them to circulate for 2 hours. Using spinning disk confocal microscopy, we could detect a signal from the OX26 immunoliposomes along the microvessels of the brain that was not readily detectable in animals injected with isotype IgG immunoliposomes (Fig. 5, left). The fluorescent signal was weak, which is consistent with earlier observations^14^. Furthermore, we also observed small particulate signals in the brain parenchyma (i.e. not associated with the brain capillaries), but these could also be detected in non-treated animals, and were therefore likely an artefact of the paraformaldehyde fixation (Fig. 5, arrows)^16^. Immunohistochemistry detecting the OX26 antibody on the immunoliposome surface was also associated with vessel-like structures that corresponded to the localization of the green fluorescent signal of the liposomes (Fig. 5). This staining could readily be observed throughout the brain tissue (Supplementary Fig. S8), but not in animals treated with isotype IgG immunoliposomes (Fig. 5). No OX26 antibody staining could be observed in areas regarded as brain parenchyma as opposed to the brain capillaries (Fig. 5 and Supplementary Fig. S8).

### Transferrin receptor-targeting increases the accumulation of platinum in the spleen and brain

**Figure 6:**
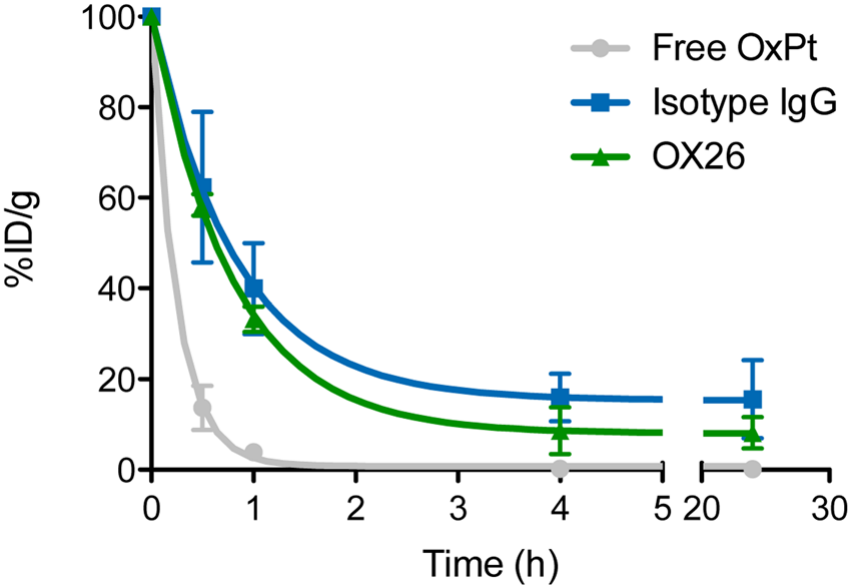
Circulation properties of immunoliposomes and free oxaliplatin. Oxaliplatin-loaded immunoliposomes or free oxaliplatin were injected into young rats and allowed to circulate. Blood was sampled at various time points, and the platinum content quantified by ICP-MS. Free oxaliplatin had poor circulation properties with only a very small amount of platinum being present in the systemic circulation after 1 hour. Encapsulation in the immunoliposomes increased the circulation time of the platinum substantially, with indications of isotype IgG immunoliposomes residing the longest in the systemic circulation. Data are presented as mean ± SD (n = 3–5). %ID/g: Percentage of injected dose per gram. OxPt: Oxaliplatin.

Since we observed that the OX26 immunoliposomes associated with the brain capillaries in the rat, but found no evidence of transcytosis into the brain parenchyma, we hypothesized that the strong association could facilitate more delivery of a small molecule cargo (oxaliplatin) encapsulated in the OX26 immunoliposomes. OX26 immunoliposomes, isotype IgG immunoliposomes or free oxaliplatin were intravenously injected into young rats (P18–P20), and blood sampled at several time points to evaluate the circulatory profile of each injected formulation in plasma (Fig. 6). Free oxaliplatin was rapidly cleared from the systemic circulation with only a very small fraction of the injected dose still present in the blood after 1 hour (<5%). The two immunoliposomal formulations had a much better circulation profile compared to the free oxaliplatin with the isotype IgG immunoliposome-associated platinum being present in the highest amount at time points later than 30 min.

**Figure 7:**
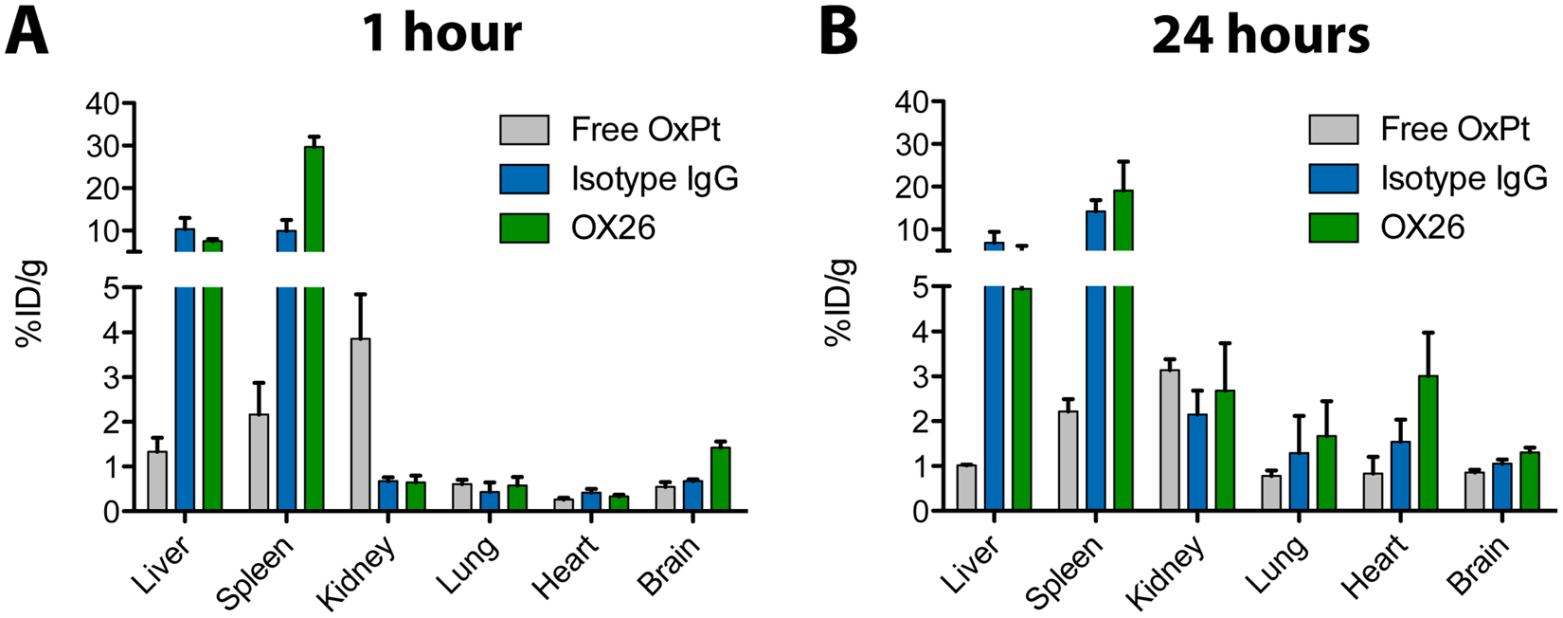
Biodistribution of oxaliplatin 1 and 24 hours after administration. Oxaliplatin-loaded immunoliposomes or free oxaliplatin were intravenously injected into young rats and allowed to circulate. At the specified time points, the rats were sacrificed and their organs resected to be analyzed for their content of platinum by ICP-MS. (**A**) After 1 hour, there was a high liver and spleen accumulation of platinum in the groups treated with immunoliposomes, with OX26 immunoliposomes having a very high uptake in the spleen. Free oxaliplatin accumulated more in the kidneys compared to the immunoliposomal formulations, whereas the OX26 immunoliposomes had a high accumulation in the brain compared to free oxaliplatin and isotype IgG immunoliposomes. (**B**) After 24 hours, there was still high accumulation of immunoliposomes in the liver and spleen, but compared to the early time point, the other peripheral organs (i.e. kidney, lung and heart) showed greater accumulation of immunoliposomes. Data are presented as mean + SEM (n = 4–5). %ID/g: Percentage of injected dose per gram. OxPt: Oxaliplatin.

Biodistribution analysis was performed to evaluate the primary accumulation sites of the different injected formulation after 1 and 24 hours. After 1 hour, OX26 immunoliposomes accumulated to a large degree in the spleen compared to isotype IgG immunoliposomes. The immunoliposomal accumulation in this organ was in general much higher than that of free oxaliplatin (Fig. 7A). No differences were found between the two immunoliposomal formulations with respect to accumulation in the liver, but there was a large difference compared to the free oxaliplatin. Free oxaliplatin accumulated more in the kidneys compared to the immunoliposomes, which corresponds well with the known clearance pathway via glomerular filtration. There was only little accumulation of the injected formulations in other organs such as the lung, heart, and brain after 1 hour, but interestingly, for the animals receiving OX26 immunoliposomes, there was an increased platinum content in the brain compared to the other formulations (Fig. 7A). After 24 hours, there was an increased accumulation of platinum in the kidney, lung, and heart compared to the early time point for the immunoliposomal formulations, while the improved brain accumulation was less pronounced (Fig. 7B).

### Transferrin receptor-targeting improves the transport of platinum into the brain parenchyma

**Figure 8:**
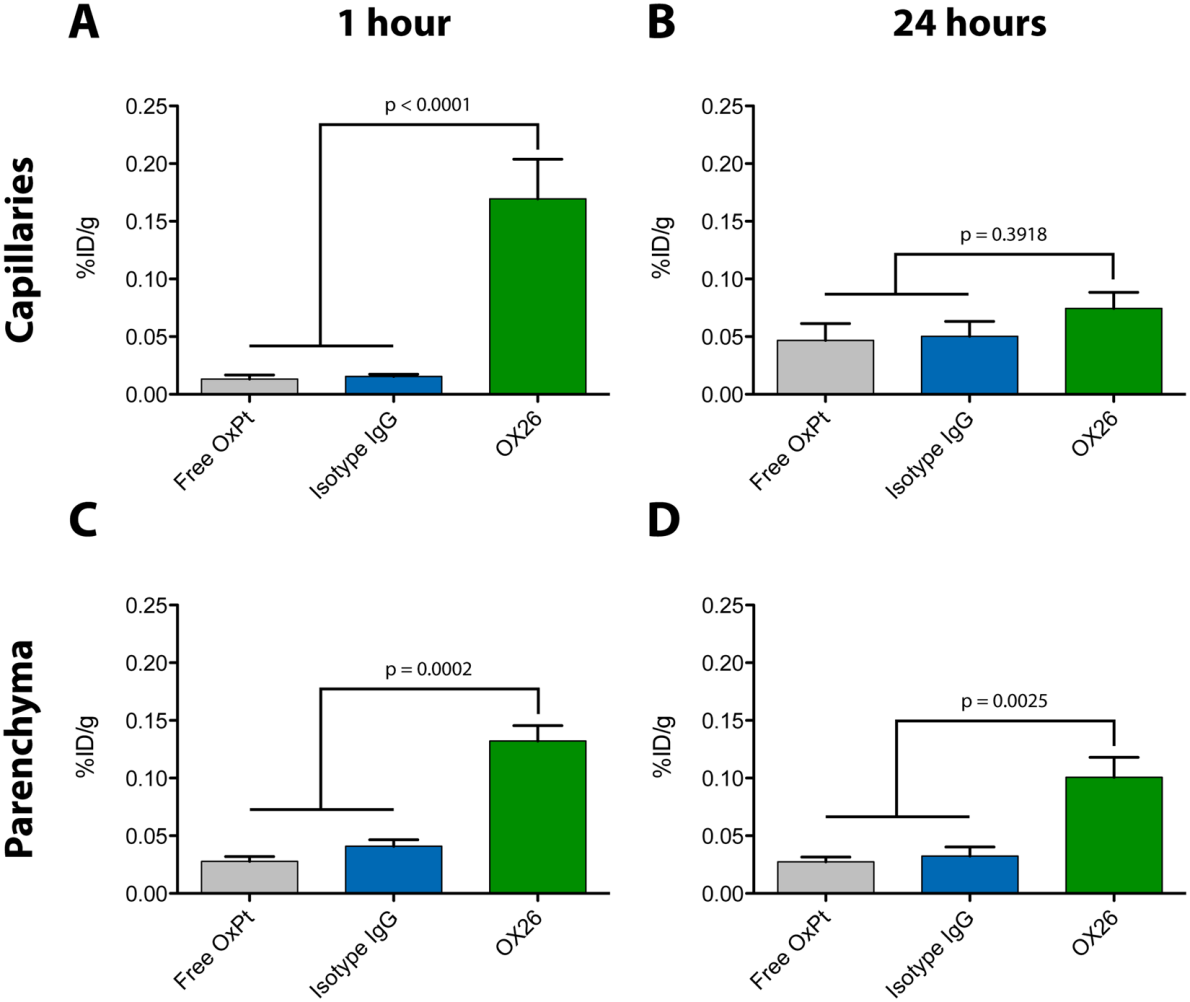
Uptake of oxaliplatin into different brain fractions after capillary depletion. Oxaliplatin-loaded immunoliposomes or free oxaliplatin were injected into young rats and allowed to circulate for the specified number of hours. The rats were sacrificed and their brains homogenized for subsequent capillary depletion. The isolated fractions from the capillary depletion (capillaries or parenchyma) were analysed for their content of platinum by ICP-MS. (**A**) After 1 hour, there was a pronounced platinum accumulation in the brain capillaries of rats treated with OX26 immunoliposomes as opposed to those treated with isotype IgG immunoliposomes or free oxaliplatin (p < 0.0001). (**B**) This difference in brain capillary accumulation between the different groups could not be detected after 24 hours (p = 0.3918). (**C**) After 1 hour, treatment with OX26 immunoliposomes facilitated a higher platinum content in the brain parenchyma compared to treatment with isotype IgG immunoliposomes or free oxaliplatin (p = 0.0002), (**D**) which seemed to stay residing after 24 hours (p = 0.0025). Data are presented as mean + SEM (n = 4–5), and the p-values depicted were derived from a one-way ANOVA with Tukey’s multiple comparisons post hoc test. %ID/g: Percentage of injected dose per gram. OxPt: Oxaliplatin.

To investigate whether the increased accumulation of platinum in the brain after treatment with OX26 immunoliposomes also correlated with increased platinum content in the brain parenchyma, we further processed the brain tissue and employed the brain capillary depletion technique to separate the capillaries from the brain parenchyma. This analysis revealed that OX26 immunoliposomes could increase the platinum content both in the brain capillaries (Fig. 8A, p < 0.0001) and in the brain parenchyma (Fig. 8C, p = 0.0002) 1 hour post injection. In the latter, the uptake of platinum increased approximately three-fold in rats receiving OX26 immunoliposomes compared to isotype IgG immunoliposomes (∼0.13%ID/g and 0.04%ID/g, respectively). After 24 hours, there was no difference in the platinum content of the brain capillaries (Fig. 8B, p = 0.3918), while ∼0.10%ID/g was still present in the brain parenchyma at this point (Fig. 8D, p = 0.0025). This suggests that active targeting against the transferrin receptor can increase the transport of a immunoliposome-encapsulated cargo without transport of the immunoliposome itself.

## Discussion

Drug delivery to the brain remains a major challenge due to the obstacles imposed by the BBB, and despite many years of research, there has only been little progress, especially in the field of nanomedicine^17^. The reason why many of the nanomedicines showing great therapeutic potential in preclinical studies fail to progress into the clinic may be multifaceted with several factors impacting on the fate of the nanomedicine strategy. These include inadequate nanocarrier design, testing in preclinical disease models that have only little correlation to the human disease, and especially the choice and function of the receptor that is targeted to increase brain exposure^12^. The transferrin receptor has been extensively studied as a potential brain drug delivery target for more than 25 years with the concept proposed even earlier by Jefferies *et al*. in 1984^6^. With respect to nanomedicine, the idea of using the transferrin receptor as a brain targeting moiety was pioneered by the studies of William Pardridge and colleagues, who in the mid-1990s showed that immunoliposomal delivery of various compounds was feasible and relied on effective transcytosis of liposomal complex and encapsulated cargo^18–25^. Using the OX26 antibody as the targeting molecule, immunoliposomes containing daunomycin as cargo were injected into rats. Following intravenous injection, daunomycin could subsequently be measured inside the brain parenchyma^18^. Intriguingly, the fraction of drug that reached the brain tissue was an order of magnitude lower than what was shown for the OX26 antibody alone^4, 18^. The study did not provide any evidence of effective BBB passage (i.e. as could be obtained by brain capillary depletion), but showed that the brain daunomycin concentration increased with varying antibody density on the liposome surface^18^. This was later accomplished; first with the same liposomal formulation after *in situ* brain perfusion, and later by intravenous injection of the immunoliposomes^26, 27^.

In the present study, we took a parallel approach by testing the transferrin receptor-targeted immunoliposomes both *in vitro* and *in vivo* for their ability of binding to BCECs and mediate transport into the brain. We chose to use the well-known antibody against the rat transferrin receptor, OX26, since it has been investigated in many studies, often with conflicting results as to the potential of this antibody to mediate transport into the brain^13, 14, 19, 28, 29^. Using fluorescently labelled immunoliposomes, we showed that transferrin receptor-targeting increased the association of the immunoliposomes with the BCECs both *in vitro* and *in vivo*. Furthermore, we showed in primary rat BCECs that this association could be inhibited both by co-incubation with free antibody and incubation at 4 °C, suggestive of an active, endocytotic uptake mechanism. The latter was in good accordance with findings obtained from *in vitro* studies using the immortal rat brain endothelial cell line, RBE4^26^. Endocytosis seemed to occur in the periphery of the cell, i.e. close to the tight junctions. Two hours after treatment, the fluorescent signal of the OX26 immunoliposomes had only weak co-localization with EEA-1, which may both be due to the time point of analysis, and that the processing for immunocytochemistry may have dissolved away some of the immunoliposomes, hereby decreasing the signal intensity. Co-localization could be detected between the OX26 immunoliposomes and LysoTracker, indicating that the immunoliposomes had been sorted towards the lysosomes after endocytosis. This observation underscores previous findings that the lysosomes are a frequent accumulation site of OX26 immunoliposomes and transferrin-decorated liposomes^30, 31^. In fact, the authors behind these findings claimed that lysosomal accumulation is a saturable process that can be overcome to facilitate transport across the BBB, and that the liposomes remain stable in the acidic compartment of the lysosomes^30, 31^.

*In vivo*, we observed that the OX26 immunoliposomes associated with the microvessels of the brain, albeit with a very weak fluorescent signal, which was also shown in a previous study^14^. Counterstaining against the OX26 antibody on the liposome surface revealed a prominent accumulation along the brain microvessels, supporting the movement of the immunoliposomes towards the brain. We could not, however, provide evidence for transcytosis of the whole immunoliposome or the ligand bound to its surface. This finding supports earlier reports showing no transcytosis of whole liposomes or free transferrin receptor antibodies binding the transferrin receptor with high affinity^13, 14, 32–34^. The ability of the transferrin receptor to transcytose through the BCEC (as an indicator for the possibility of whole nanoparticle transcytosis) has remained an area of dispute for many years^10^. Some groups have argued for a transcytosis mechanism largely based on studies of radiolabelled OX26 antibodies and transferrin^35^, whereas others claim this to be impossible, since rats with a mutant DMT1 transporter take up less iron into the brain^5^. In addition, recent studies have shown that BCECs contain all the necessary machinery for an iron uptake mechanism based on endocytosis and dissociation of the iron atom from the transferrin receptor-transferrin complex, hereby suggesting that the transcytosis pathway must be of very little importance, if existing at all^7–10^. This greatly impact the way we interpret findings where transferrin receptor-targeted drug constructs or nanomedicines provide favourable therapeutic outcomes in disease models, or simply increase the amount of drug exposure in the brain. Still, it is important to note that some groups working with inorganic, transferrin receptor-targeted nanoparticles have observed transport of whole nanoparticles into the brain *in vivo* ^36–38^. Conversely, new interesting findings indicate that retrograde receptor, like the mannose-6-phosphate receptor 300 (MPR300), might be more efficient in performing direct transcytosis^39^.

Since we did not prove transport of whole immunoliposomes across the BBB, we hypothesized that the strong association between the OX26 immunoliposomes and BCECs both *in vitro* and *in vivo* could be exploited to increase the brain uptake of a small molecule drug. We chose to work with the chemotherapeutic drug, oxaliplatin, because it contained a platinum atom that could be detected with great sensitivity using ICP-MS. Hereby, we could avoid the problems that the use of a fluorescently labelled compound would have posed in quantitative analyses^40^. When administrated to the primary rat *in vitro* model of the BBB, OX26 immunoliposomes mediated an increased uptake of platinum into the BCECs compared to isotype IgG immunoliposomes, although the difference was not as pronounced as observed in flow cytometry. Interestingly, we did not find that OX26 immunoliposomes improved the transport of platinum across the BCECs *in vitro*, and in fact, isotype IgG immunoliposomes performed significantly better in these experiments. This outcome may be attributed to the long incubation period that was used for these experiment, since this was needed to measure sufficient amounts of platinum in the ICP-MS analysis. Thus, a longer exposure of the BCECs to the immunoliposomes may have allowed for some unspecific interaction that improved the transport in the isotype IgG immunoliposome group. Furthermore, transcytosis studies in the Transwell co-culture system does not account for the flow conditions that would be present *in vivo*, which would likely decrease the level of unspecific interaction^41^. Others have raised concern about the use of Transwell co-cultures for assessment of transcytosis abilities of drug constructs and nanomedicines, since these models may have several caveats that pertain to the use of immortal brain endothelial cells lines and their lack of ability to form a tight barrier, or because the filters of the Transwell insert will likely interact with any transcytosed material^41, 42^. To our knowledge, our study is among the very few to include an *in vitro* model of the BBB based on primary cells for investigations of transferrin receptor-targeted nanoparticles. Other issues may be controlled by employing the recently described pulse-chase technique for assessment of transcytosis^43^. We have recently shown that the primary rat *in vitro* BBB employed in this study possessed all the necessary machinery for mediating transferrin receptor-mediated uptake followed iron transport into the brain via the ‘DMT1 pathway’. Hence, we believe that this model is fit to study transferrin receptor-targeting as well^9^. Still, *in vitro* data should always be interpreted with caution when attempting to generalize the findings to the *in vivo* setting.

Injection of oxaliplatin-loaded OX26 immunoliposomes mediated a higher uptake into the brain after 1 hour compared to both isotype IgG immunoliposomes and free oxaliplatin. The observation was further substantiated by the fact that the subsequent brain capillary depletion revealed that the differences could also be observed in the brain parenchyma. This suggests that transferrin receptor-targeting will facilitate preferential accumulation of the injected compound in the brain capillaries, wherefrom a yet unknown mechanism releases the small molecule cargo into the brain independently of the movement of the whole immunoliposome itself. Whether this mechanism is unspecific, i.e. releasing equal amounts of platinum to both the blood and the brain side of the BBB, we cannot decipher from these results. We speculate that the transferrin receptor-targeting may have increased the chance of fusion between the liposome and the BCEC membrane, as was recently suggested as the primary interaction outcome between a liposome and a BCEC^44^. The amount of platinum transported into the brain parenchyma (∼0.13%ID/g) falls in the high range compared to other studies of the kind (for details please refer to Johnsen & Moos (2016)^4^), although well below studies using a dual-targeting strategy by combining transferrin receptor-targeting with cationic peptides^45^. These values will of course largely depend on the type of compound that is being analyzed as a measure for transport across the BBB, which in previous studies included both radiolabelled cholesterol and daunomycin^18, 27^. Even though the brain capillary depletion technique is a valid way of separating and analysing the vascular and parenchymal compartments independently, it has several flaws that needs to be considered when interpreting the data^13, 40^. A small amount of the capillaries may stay in the parenchymal fraction, or the compound analysed may be able to freely diffuse before separation of the fractions^40^. Thus, the values obtained in brain capillary depletion experiments should only be regarded as approximations, and the main arguments drawn from these studies should be evidence of transport into the brain parenchyma and the differences observed between the individual groups studied. Furthermore, the fact that this study used young rats may also have had an impact for the observed increase in transport (due to the higher expression of transferrin receptors)^13^.

In conclusion, transferrin receptor-targeting is a robust strategy to increase immunoliposome accumulation in the BCECs of the BBB. A part of the immunoliposomes interacting with the BCEC surface seemed to endocytose into the cell, where they were sorted towards the lysosomes. Transcytosis of whole immunoliposomes could not be evidenced from this study, but the transferrin receptor-targeted immunoliposomes could increase the transport of a small molecule drug cargo into the brain parenchyma. Additional studies are needed to shed light on the possibility of full nanoparticle transcytosis into the brain via the transferrin receptor, e.g. by using inorganic nanoparticles that are not degraded in a biological setting.

## Materials and Methods

A detailed *Materials and methods* section is attached a Supplementary Information in the online version of this paper. According to the data availability policy of *Scientific Reports*, the data presented in this paper is available upon reasonable request.

### Uptake studies evaluated by flow cytometry

To evaluate association and/or uptake of fluorescently labelled immunoliposomes into BCECs, *in vitro* BBB models were treated with either OX26 immunoliposomes, isotype IgG immunoliposomes or stealth liposomes (with no antibodies attached) (60 nmol lipid per well) and incubated for 2 hours. The cells were washed carefully three times using 0.1 mg/mL heparin in 0.1 M PBS (pH 7.4) and then detached from the culture insert membranes using trypsin. The trypsinized cells were transferred to flow cytometry tubes and centrifuged at 140 rcf for 5 min and resuspended in 0.1 M PBS (pH 7.4). The amount of association between immunoliposomes and BCECs were measured on a MoFlo® Flow Cytometer System™ (Beckman-Coulter, Copenhagen, DK). Prior to analysis, the flow cytometer parameters were calibrated using SPHERO™ Ultra Rainbow Fluorescent Particles (3 µm)(Spherotech, Lake Forrest, IL, USA). Cells were gated based on the forward/side scatter plot to eliminate cell debris in the subsequent analysis. The fluorescence intensity of the immunoliposome-treated cells was corrected based on gating of the autofluorescence of non-treated cells. The resulting data was analyzed using Kaluza software (Beckman-Coulter, Copenhagen, DK), and the median fluorescence intensity of the individual treatment groups was plotted in GraphPad Prism 5.0 (GraphPad Software, Inc., CA, USA).

Each preparation of fluorescently labelled immunoliposomes were analyzed for their binding capacity to the immortalized rat brain endothelial cell line, RBE4, prior to any experiments using primary rat brain endothelial cells. The cells were seeded in collagen I-coated 12-well plates and grown to a monolayer in Alpha-MEM + Ham’s F10 (Thermo Scientific, Hvidovre, DK) with 10% FCS, 300 µg/mL G418 (Lonza Copenhagen, Vallensbæk Strand, DK) and 1 ng/mL bFGF (Roche, Hvidovre, DK) before administration of fluorescently labelled immunoliposomes in the concentration described above. After incubation, the cells were washed and trypsinized as described above, and analyzed by flow cytometry using a Gallios^TM^ Flow Cytometer (Beckman-Coulter, Copenhagen, DK). To investigate the possible cross-reactivity of the prepared immunoliposomes to the mouse transferrin receptor, the immortalized mouse brain endothelial cell line, bEnd.3, was included in experiments like those above (Supplementary Fig. S4). The bEnd.3 cells were cultured in DMEM containing 10% FCS and 1% penicillin and streptomycin.

### Uptake and transcytosis studies using oxaliplatin-encapsulated immunoliposomes

After reaching high TEER (regarded as >150 Ω*cm^2^ as suggested by Burkhart *et al*.^15^), the BCECs were treated with oxaliplatin-loaded immunoliposomes (OX26 or isotype IgG) at a lipid concentration of 120 µM corresponding to total amount of 2.65 µg oxaliplatin per insert. Immediately after the administration, a sample was taken from each insert to measure the exact platinum content administered. The BCECs were incubated for 4 hours to allow for liposome/oxaliplatin uptake and transport across the membrane. The TEER was measured again after the incubation to ensure the barrier integrity was maintained during the experiment (Supplementary Fig. S5). Afterwards, samples were taken from the culture medium in the insert (‘blood fraction’) and the bottom well (‘brain fraction’, see Fig. 4A). The BCECs in the insert were carefully washed three times using 0.1 mg/mL heparin in 0.1 M PBS (pH 7.4) and detached from the culture insert membranes using trypsin (‘BCEC fraction’). The platinum content in each sample was measured using ICP-MS (see below), and the resulting data was analysed in GraphPad Prism 5.0 (GraphPad Software, Inc., CA, USA). The results for platinum uptake and transport was plotted as percentage of administrated dose (%AD) as depicted from the sample taken immediately after administration.

### *In vivo* studies and tissue isolation

All procedures and handling of rats were approved by the Danish National Council for Animal Welfare (License no. 2013-15-2934-00893). The experiments were all performed in accordance with relevant guidelines and regulations (EU directive 2010/63/EU and relevant local additions to this directive). Uptake studies *in vivo* were performed using male P18-P20 Sprague-Dawley rats (n = 5 per group), which were intravenously injected through the lateral tail vein with fluorescently labelled immunoliposomes for morphological studies, or oxaliplatin-loaded immunoliposomes or free oxaliplatin for brain uptake and biodistribution studies.

For morphological analysis, fluorescently labelled immunoliposomes were injected (5 µmol lipid per animal) and allowed to circulate for 2 hours. Afterwards, the rats were deeply anesthetized by a subcutaneous injection of 0.5 ml/10 g body weight of Hypnorm/Dormicum (fentanyl/fluanisone mixed with midazolam and sterile water in a ratio of 1:1:2). The chest cage was opened, and the rats transcardially perfused with 0.1 M PBS (pH 7.4) followed by 4% paraformaldehyde in 0.1 M PBS (pH 7.4). The fixed rats were decapitated and the skull opened to expose the brain. The brains were submerged into fixative for 24 hours followed by extensive washing in KPBS and immersion in 30% sucrose. Brains were then sectioned into 40 µm slices and kept at −20 °C in anti-freeze solution until further processing.

For quantitative brain uptake and biodistribution studies, the oxaliplatin-loaded immunoliposomes (5 µmol lipid per animal corresponding to 186 µg oxaliplatin) or free oxaliplatin (186 µg per animal) were injected, and allowed to circulate for 0, 0.5, 1, 4, and 24 hours. After the circulation, the rats were deeply anesthetized by a subcutaneous injection of 0.5 ml/10 g body weight of Hypnorm/Dormicum (fentanyl/fluanisone mixed with midazolam and sterile water in a ratio of 1:1:2). The chest cage was opened, and the rats were transcardially perfused with 0.1 M PBS (pH 7.4). After perfusion, the skull was opened and the brains removed. One hemisphere of the brain was quickly frozen on dry ice, and the other used for brain capillary depletion (see below). Tissue samples were also taken from the liver, spleen, kidney, lung and heart, which were quickly frozen on dry ice too and stored until processing for ICP-MS.

Before perfusion of the animals with 0.1 M PBS (pH 7.4), blood samples were drawn from the left ventricle of the heart into a heparin-containing tube (time points: 0, 0.5, 1, 4, and 24 hours). The blood samples were centrifuged at 2,000 rcf for 15 min to pellet cells and platelets, and the plasma transferred into clean tubes and stored at −20 °C. The platinum content was measured using ICP-MS (see below) and the resulting data plotted as %ID/g in GraphPad Prism 5.0 (GraphPad Software, Inc., CA, USA).

### Brain capillary depletion

The presence of transcytosed platinum in the brain parenchyma was evaluated by separating the brain capillaries using the brain capillary depletion technique of Triguero *et al*. (1990) with minor modifications^46^. One hemisphere per rat was homogenized by four strokes in a dounce homogenizer in 3.5 mL ice-cold homogenization buffer (10 mM HEPES, 141 mM NaCl, 4 mM KCl, 2.8 mM CaCl_2_, 1 mM MgSO_4_, 1 mM NaH_2_PO_4_, and 10 mM glucose; pH 7.4). The homogenized brain tissue was mixed with 3.5 mL ice-cold 30% dextran (MW: 60,000, Sigma-Aldrich), and the mixture was further homogenized by four strokes. The homogenate was then transferred to a 15 mL Falcon tube and centrifuged at 3,500 rcf for 40 min at 4 °C with slow deceleration. Afterwards, the supernatant was removed and stored separately from the capillary-containing pellet. Samples were taken from both fractions and processed for ICP-MS to quantify the amount of platinum (see below). The purity of the depleted capillaries was determined by the expression of alkaline phosphatase, and corresponded to what was previously shown in the rat^13, 14^.

### Inductively-coupled plasma mass spectrometry

To analyse the platinum content in the extracted tissue samples, a maximum of 100 mg tissue was digested in aqua regia overnight at 65 °C. After complete digestion, the samples were diluted in MilliQ water containing 0.5 ppb iridium (Fluka, Sigma-Aldrich, Brøndby, DK) followed by dilution in 2% HCl containing 0.5 ppb iridium. All samples were analyzed on a iCAP Q ICP-MS system (Thermo Scientific, Hvidovre, DK) fitted with an ASX-520 AutoSampler and a Neclar ThermoFlex 2500 chiller. Prior to analysis, the instrument was calibrated using TUNE B iCAP Q element mixture (Thermo Scientific, Hvidovre, DK), and a standard curve was generated based on serial dilution of an analytical standard platinum solution (Fluka, Sigma-Aldrich, Brøndby, DK) to obtain data points ranging from 0.08–10 ppb. In addition to analysing the platinum content in each sample, the iridium content was measured as an internal standard. The resulting data was plotted as %ID/g in GraphPad Prism 5.0 (GraphPad Software, Inc., CA, USA).

To measure the content of phosphor in the liposome formulations (as an indicator of phospholipid concentration), a sample of liposomes was diluted in 2% HCl containing 0.5 ppb gallium (Fluka, Sigma-Aldrich, Brøndby, DK). All samples were analysed as described above with a standard curve generated from serial dilution of an analytical standard phosphor solution (Fluka, Sigma-Aldrich, Brøndby, DK).

## Electronic supplementary material

Supplementary information

## References

[1] Abbott NJ (2013). Blood-brain barrier structure and function and the challenges for CNS drug delivery. J. Inherit. Metab. Dis..

[2] Zlokovic BV (2008). The blood-brain barrier in health and chronic neurodegenerative disorders. Neuron.

[3] Saraiva C (2016). Nanoparticle-mediated brain drug delivery: Overcoming blood-brain barrier to treat neurodegenerative diseases. J Control Release.

[4] Johnsen KB, Moos T (2016). Revisiting nanoparticle technology for blood-brain barrier transport: Unfolding at the endothelial gate improves the fate of transferrin receptor-targeted liposomes. J Control Release.

[5] Leitner DF, Connor JR (2012). Functional roles of transferrin in the brain. Biochim. Biophys. Acta.

[6] Jefferies WA (1984). Transferrin receptor on endothelium of brain capillaries. Nature.

[7] Simpson IA (2015). A novel model for brain iron uptake: introducing the concept of regulation. J. Cereb. Blood Flow Metab..

[8] Duck KA, Connor JR (2016). Iron uptake and transport across physiological barriers. Biometals.

[9] Burkhart A (2016). Expression of Iron-Related Proteins at the Neurovascular Unit Supports Reduction and Reoxidation of Iron for Transport Through the Blood-Brain Barrier. Mol Neurobiol.

[10] Skjørringe T, Burkhart A, Johnsen KB, Moos T (2015). Divalent metal transporter 1 (DMT1) in the brain: implications for a role in iron transport at the blood-brain barrier, and neuronal and glial pathology. Front Mol Neurosci.

[11] Freskgård, P.-O. & Urich, E. Antibody therapies in CNS diseases. *Neuropharmacology*, doi:10.1016/j.neuropharm.2016.03.014 (2016). 10.1016/j.neuropharm.2016.03.014 26972827

[12] Johnsen, K. B., Burkhart, A., Andresen, T. L., Moos, T. & Thomsen, L. B. In Na*nom*edicine*s* for B*rain Drug Delivery* (eds Gaillard, P. J. & Morales, J.) (Springer, 2017).

[13] Moos T, Morgan EH (2001). Restricted transport of anti-transferrin receptor antibody (OX26) through the blood-brain barrier in the rat. J. Neurochem..

[14] Gosk S, Vermehren C, Storm G, Moos T (2004). Targeting anti-transferrin receptor antibody (OX26) and OX26-conjugated liposomes to brain capillary endothelial cells using *in situ* perfusion. J. Cereb. Blood Flow Metab..

[15] Burkhart A (2015). Transfection of brain capillary endothelial cells in primary culture with defined blood-brain barrier properties. Fluids Barriers CNS.

[16] Spitzer N, Sammons GS, Price EM (2011). Autofluorescent cells in rat brain can be convincing impostors in green fluorescent reporter studies. J. Neurosci. Methods.

[17] Fullstone G, Nyberg S, Tian X, Battaglia G (2016). From the Blood to the Central Nervous System: A Nanoparticle’s Journey Through the Blood-Brain Barrier by Transcytosis. Int. Rev. Neurobiol..

[18] Huwyler J, Wu D, Pardridge WM (1996). Brain drug delivery of small molecules using immunoliposomes. Proc. Natl. Acad. Sci. U.S.A..

[19] Shi N, Pardridge WM (2000). Noninvasive gene targeting to the brain. Proc. Natl. Acad. Sci. USA.

[20] Shi N, Zhang Y, Zhu C, Boado RJ, Pardridge WM (2001). Brain-specific expression of an exogenous gene after i.v. administration. Proc. Natl. Acad. Sci. USA.

[21] Shi N, Boado RJ, Pardridge WM (2001). Receptor-mediated gene targeting to tissues *in vivo* following intravenous administration of pegylated immunoliposomes. Pharm. Res..

[22] Huwyler J, Cerletti A, Fricker G, Eberle AN, Drewe J (2002). By-passing of P-glycoprotein using immunoliposomes. J Drug Target.

[23] Zhang Y-F, Boado RJ, Pardridge WM (2003). Absence of toxicity of chronic weekly intravenous gene therapy with pegylated immunoliposomes. Pharm. Res..

[24] Zhang Y, Schlachetzki F, Zhang Y-F, Boado RJ, Pardridge WM (2004). Normalization of striatal tyrosine hydroxylase and reversal of motor impairment in experimental parkinsonism with intravenous nonviral gene therapy and a brain-specific promoter. Hum. Gene Ther..

[25] Zhang Y (2004). Intravenous RNA interference gene therapy targeting the human epidermal growth factor receptor prolongs survival in intracranial brain cancer. Clin. Cancer Res..

[26] Cerletti A, Drewe J, Fricker G, Eberle AN, Huwyler J (2000). Endocytosis and transcytosis of an immunoliposome-based brain drug delivery system. J Drug Target.

[27] van Rooy I, Mastrobattista E, Storm G, Hennink WE, Schiffelers RM (2011). Comparison of five different targeting ligands to enhance accumulation of liposomes into the brain. J Control Release.

[28] Friden PM (1991). Anti-transferrin receptor antibody and antibody-drug conjugates cross the blood-brain barrier. Proc. Natl. Acad. Sci. USA.

[29] Lichota J, Skjørringe T, Thomsen LB, Moos T (2010). Macromolecular drug transport into the brain using targeted therapy. J. Neurochem..

[30] Markoutsa E (2011). Uptake and permeability studies of BBB-targeting immunoliposomes using the hCMEC/D3 cell line. Eur J Pharm Biopharm.

[31] Gao J-Q (2013). Glioma targeting and blood-brain barrier penetration by dual-targeting doxorubincin liposomes. Biomaterials.

[32] Paris-Robidas S, Brouard D, Emond V, Parent M, Calon F (2016). Internalization of targeted quantum dots by brain capillary endothelial cells *in vivo*. J. Cereb. Blood Flow Metab..

[33] Alata W, Paris-Robidas S, Emond V, Bourasset F, Calon F (2014). Brain uptake of a fluorescent vector targeting the transferrin receptor: a novel application of *in situ* brain perfusion. Mol. Pharm..

[34] Yu YJ (2011). Boosting brain uptake of a therapeutic antibody by reducing its affinity for a transcytosis target. Sci Transl Med.

[35] Skarlatos S, Yoshikawa T, Pardridge WM (1995). Transport of [125I]transferrin through the rat blood-brain barrier. Brain Research.

[36] Cabezón I (2015). Trafficking of Gold Nanoparticles Coated with the 8D3 Anti-Transferrin Receptor Antibody at the Mouse Blood-Brain Barrier. Mol. Pharm..

[37] Wiley DT, Webster P, Gale A, Davis ME (2013). Transcytosis and brain uptake of transferrin-containing nanoparticles by tuning avidity to transferrin receptor. Proc. Natl. Acad. Sci. USA.

[38] Clark, A. J. & Davis, M. E. Increased brain uptake of targeted nanoparticles by adding an acid-cleavable linkage between transferrin and the nanoparticle core. *Proc. Natl. Acad. Sci. USA* 201517048 1517048112, doi:10.1073/pnas.1517048112 (2015). 10.1073/pnas.1517048112 PMC4603510 26392563

[39] Siupka, P. *et al*. Bidirectional apical-basal traffic of the cation-independent mannose-6-phosphate receptor in brain endothelial cells. *J. Cereb. Blood Flow Metab*. 271678X17700665, doi:10.1177/0271678X17700665 (2017). 10.1177/0271678X17700665 PMC5531359 28337939

[40] Freskgård, P. O., Niewoehner, J. & Urich, E. *Time to open the blood-brain barrier gate for biologics?* doi:10.2217/fnl.14.15 (Future Neurology, 2014).

[41] Helms HC (2016). *In vitro* models of the blood-brain barrier: An overview of commonly used brain endothelial cell culture models and guidelines for their use. J. Cereb. Blood Flow Metab..

[42] Berg C (2016). Quantitative analysis of nanoparticle transport through *in vitro* blood-brain barrier models. Tissue Barriers.

[43] Sade H (2014). A human blood-brain barrier transcytosis assay reveals antibody transcytosis influenced by pH-dependent receptor binding. PLoS ONE.

[44] Lindqvist A, Fridén M, Hammarlund-Udenaes M (2016). Pharmacokinetic considerations of nanodelivery to the brain: Using modeling and simulations to predict the outcome of liposomal formulations. Eur J Pharm Sci.

[45] Sharma G (2013). Cell penetrating peptide tethered bi-ligand liposomes for delivery to brain *in vivo*: Biodistribution and transfection. J Control Release.

[46] Triguero D, Buciak J, Pardridge WM (1990). Capillary depletion method for quantification of blood-brain barrier transport of circulating peptides and plasma proteins. J. Neurochem..

