# Supplementary material for: Targeting transferrin receptors at the blood-brain barrier improves the uptake of immunoliposomes and subsequent cargo transport into the brain parenchyma
Source: Sci Rep. 2017 Sep 4;7:10396. doi: 10.1038/s41598-017-11220-1 (PMC5583399; doi:10.1038/s41598-017-11220-1)
Supplement: Supplementary file 1 — Supplementary information [file 41598_2017_11220_MOESM1_ESM.pdf]

Supplementary figures for “Targeting transferrin receptors at the blood-brain barrier improves the uptake of immunoliposomes and subsequent cargo transport into the brain parenchyma”

Kasper Bendix Johnsen[1,2], Annette Burkhart[1], Fredrik Melander[2], Paul Joseph Kempen[2], Jonas Bruun Vejlebo[2], Piotr Siupka[3], Morten Schallburg Nielsen[3], Thomas Lars Andresen[2] & Torben Moos[1]*

[1]Laboratory for Neurobiology, Biomedicine, Institute of Health Science and Technology, Aalborg University, Aalborg, Denmark. [2]Center for Nanomedicine and Theranostics, Department of Micro- and Nanotechnology, Technical University of Denmark, Denmark. [3]Department of Biomedicine, Aarhus University, Denmark.

Supplementary Figure 1. Ellman's reaction plot depicting the correlation between molar ratio of 2-iminothiolane:antibody and the number of thiols per antibody. Ab: Antibody.

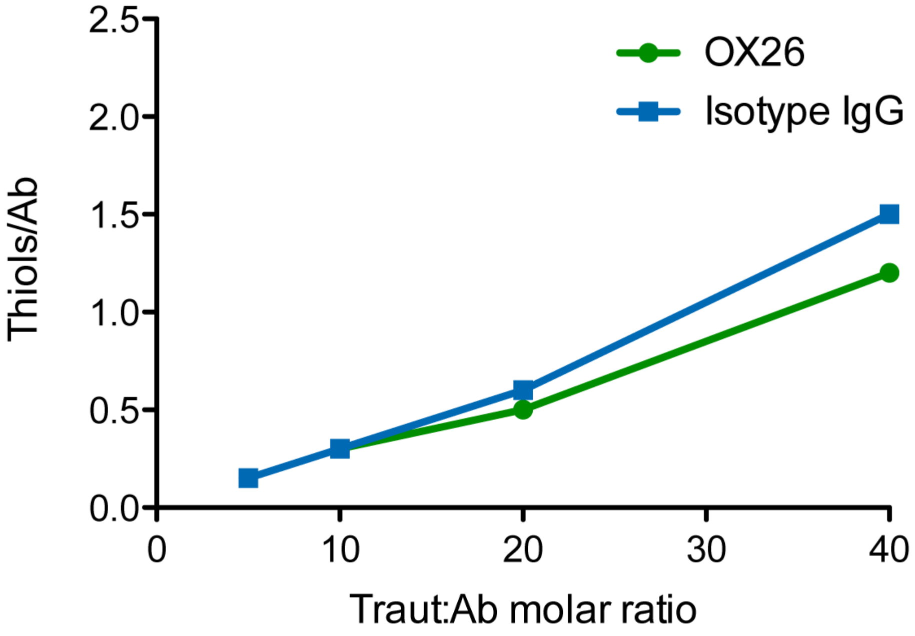

Supplementary Figure 2. Representative size exclusion chromatograms of micelles conjugated to either OX26 (A) or isotype IgG (B) antibodies based on the protein concentration. The fractions contained under the green overlay were isolated and upconcentrated.

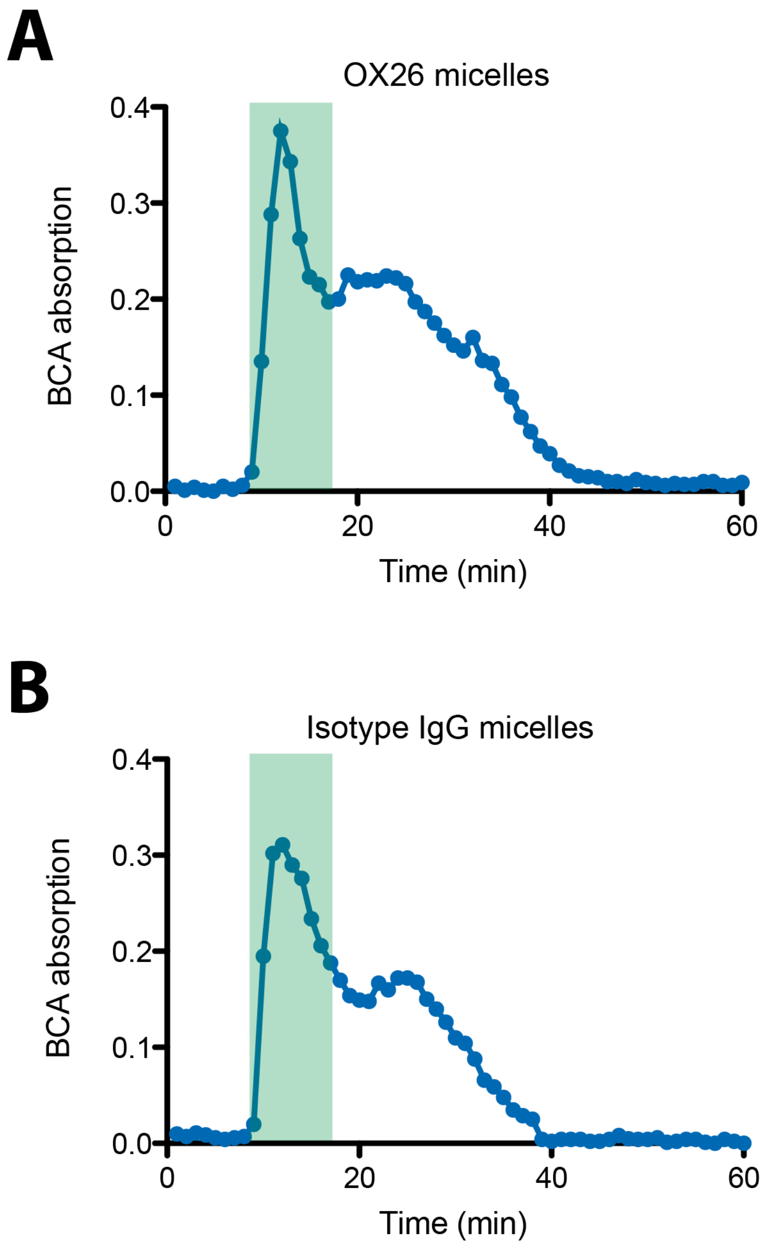

Supplementary Figure 3. Representative size exclusion chromatograms of liposomes conjugated to either OX26 (A) or isotype IgG (B) antibodies based on the protein concentration and the fluorescence intensity of the liposomes. The fractions contained under the green overlay were isolated and upconcentrated.

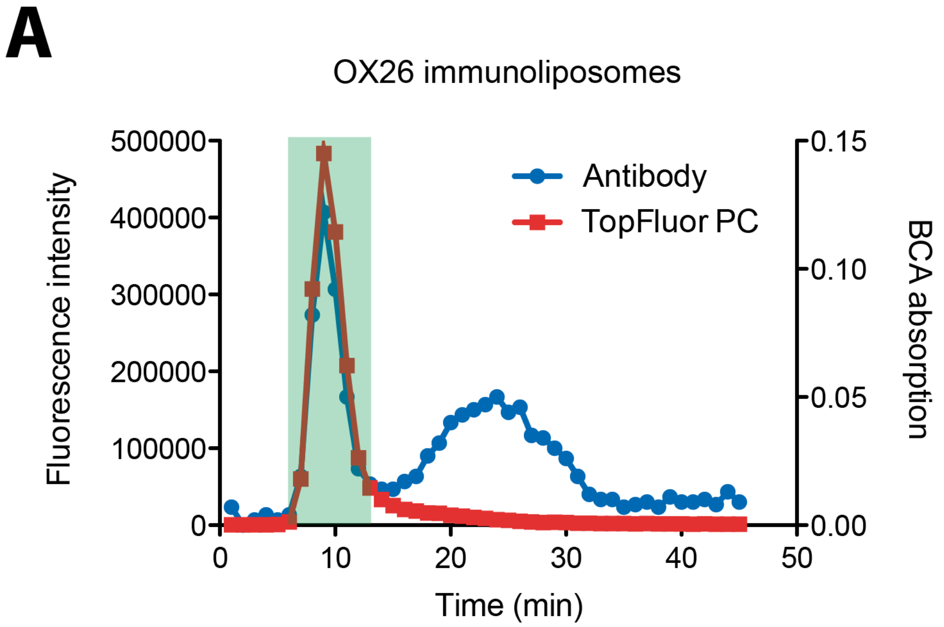

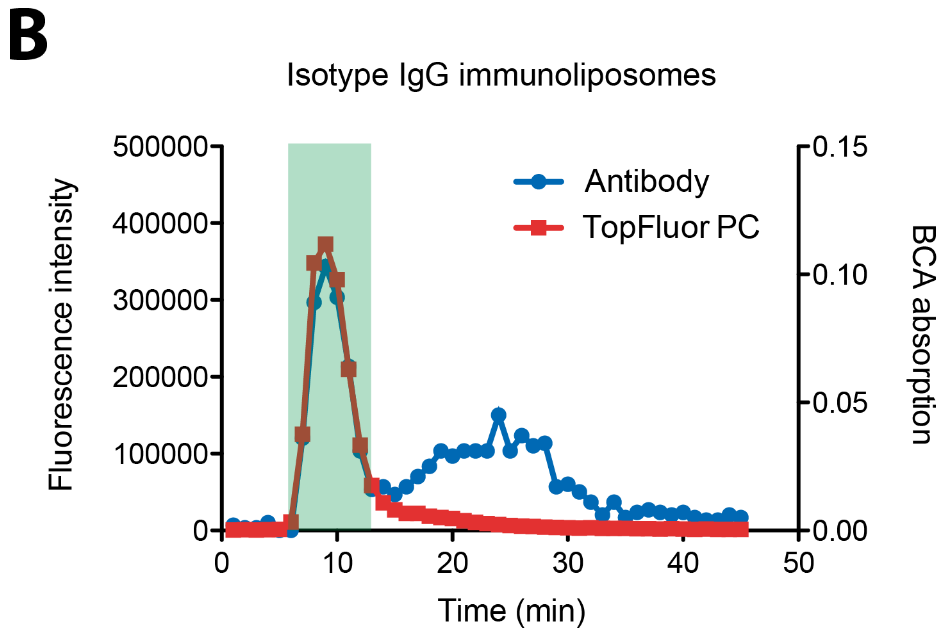

Supplementary Figure 4. Flow cytometry study of immunoliposome uptake in the RBE4 and bEnd.3 immortalized brain endothelial cell lines. The OX26 immunoliposomes associated only weakly with the mouse bEnd.3 cells compared with isotype IgG immunoliposomes. Data are presented as mean + SEM (n = 3 – 4), and the p-values depicted were derived from a one-way ANOVA with Tukey's multiple comparisons post hoc test. MFI: Median fluorescent intensity.

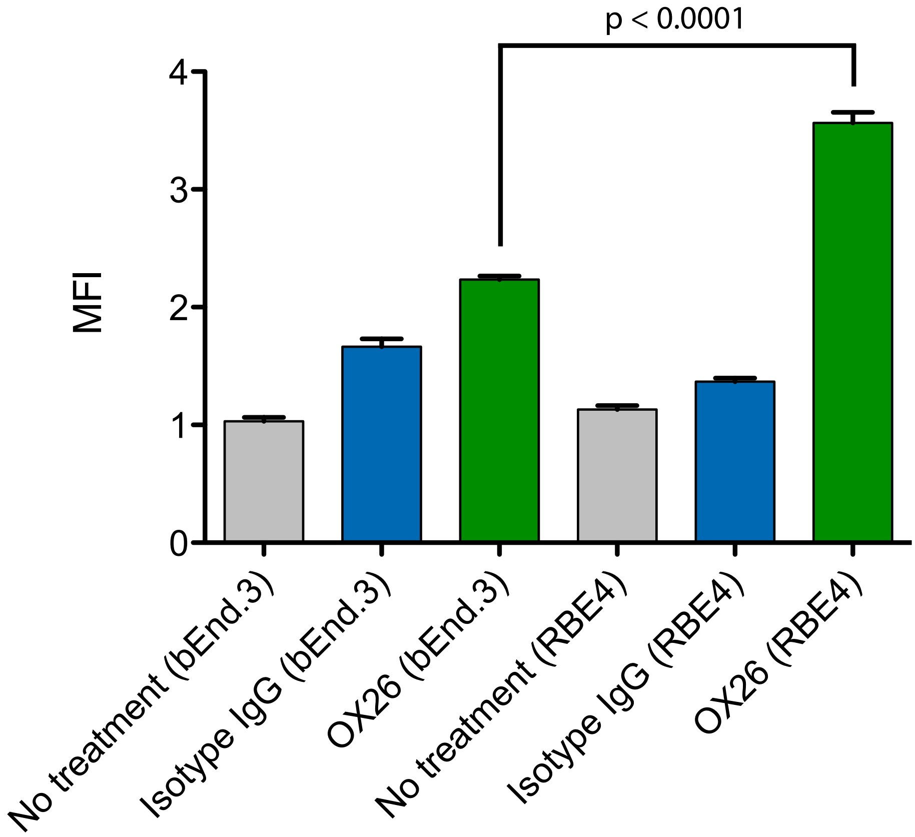

Supplementary Figure 5. TEER measurements before and after treatment with immunoliposomes. No reduction in the barrier integrity could be observed during the course of the immunoliposome treatment. TEER: Transendothelial electrical resistance.

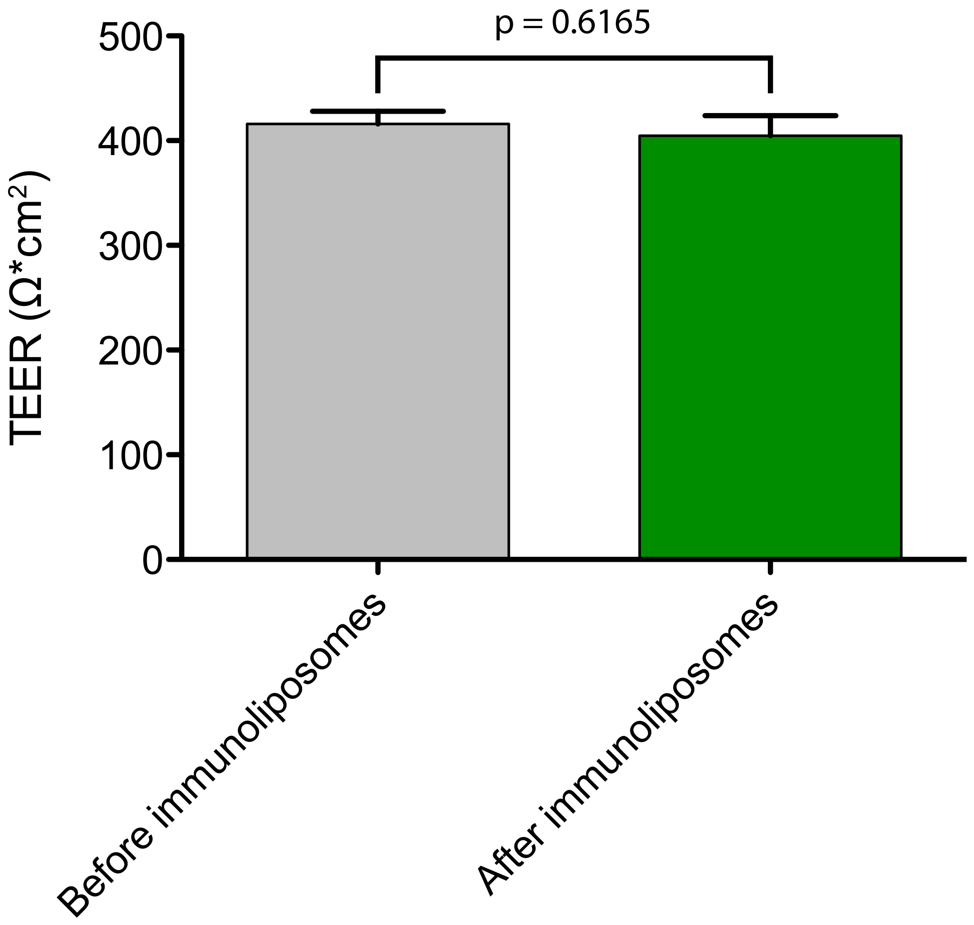

Supplementary Figure 6. Stability and morphology of oxaliplatin-loaded immunoliposomes. (A) After post-insertion of antibody micelle into the membrane of oxaliplatin-loaded liposomes, the oxaliplatin leakage was found to be minor. Data is represented as mean + SD (n = 3). (B) Although only fresh batches of oxaliplatin-loaded liposomes were used for post-insertion of antibodies, analysis of older batches revealed that even long-term storage does not result in any appreciable oxaliplatin leakage. (C+D) Representative cryo-transmission electron microscopy images of the oxaliplatin-loaded liposomes revealing that the liposomes are unilamellar and spheroid-shaped. Artefacts in the form of ice crystals can also be observed (arrows). Scale bar depicts 100 nm.

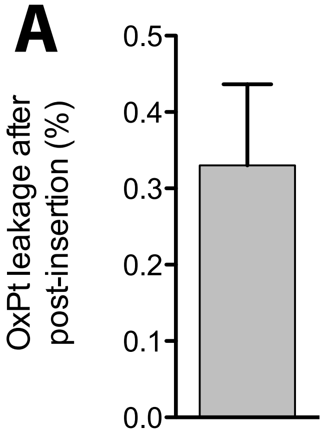

B

Age	*OxPt leakage*
New batch	*0.5 %*
4 months	*1.5 %*
12 months	*0.9 %*
24 months	*1.1 %*

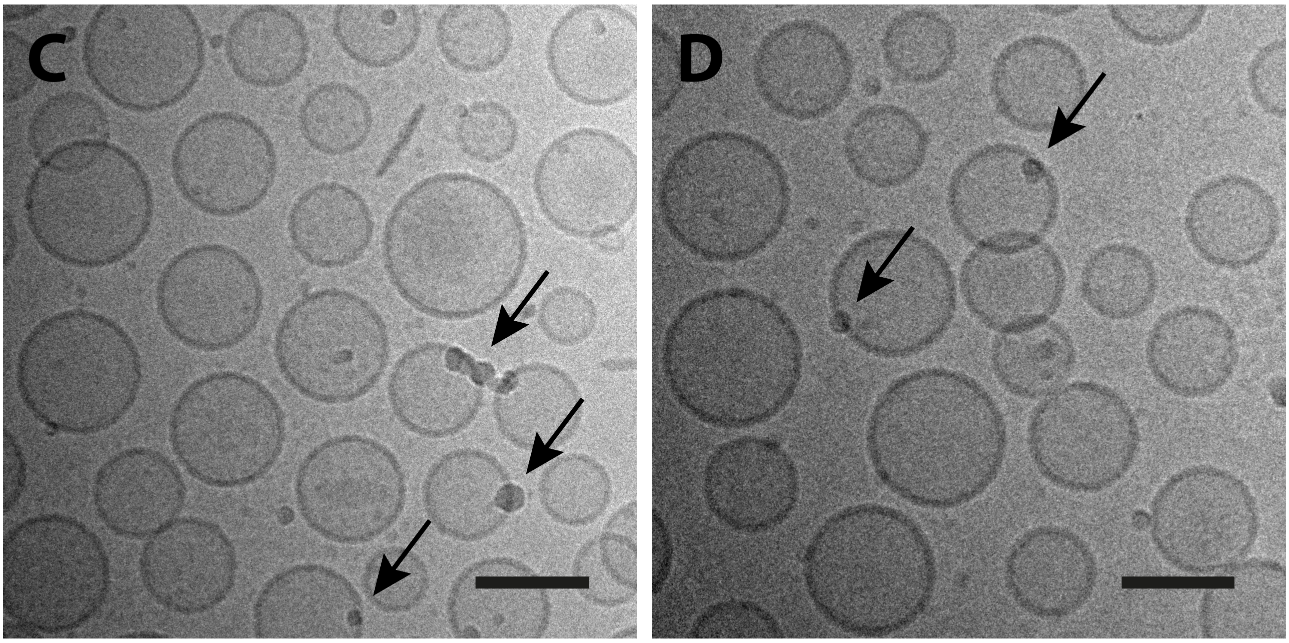

Supplementary Figure 7. Immunocytochemistry against EEA-1 and co-localization with OX26 immunoliposomes. Only weak co-localization could be observed between OX26 immunoliposomes and early endosomes.

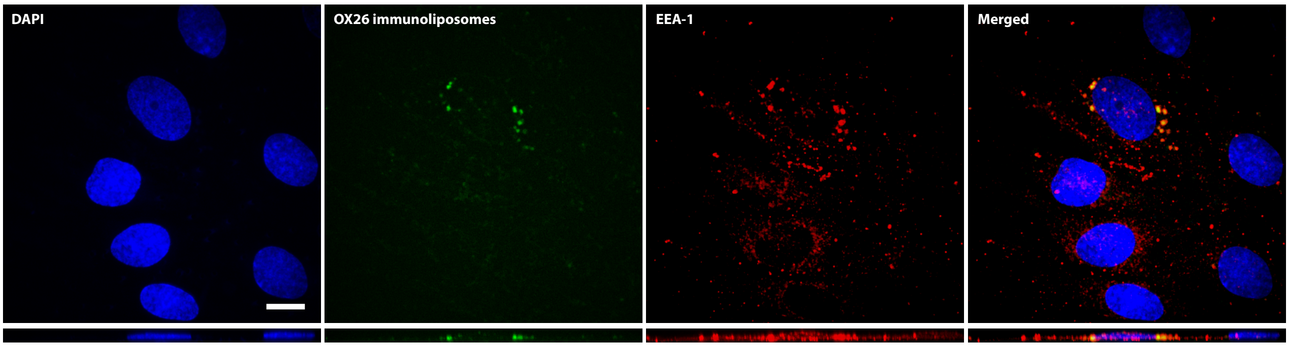

Supplementary Figure 8. Anti-OX26 staining in on rat brain sections. Staining against the OX26 antibody on the surface of the OX26 immunoliposomes revealed a clear vessel pattern in the brain, indicating accumulation at the brain capillaries, but no transcytosis of the ligand.

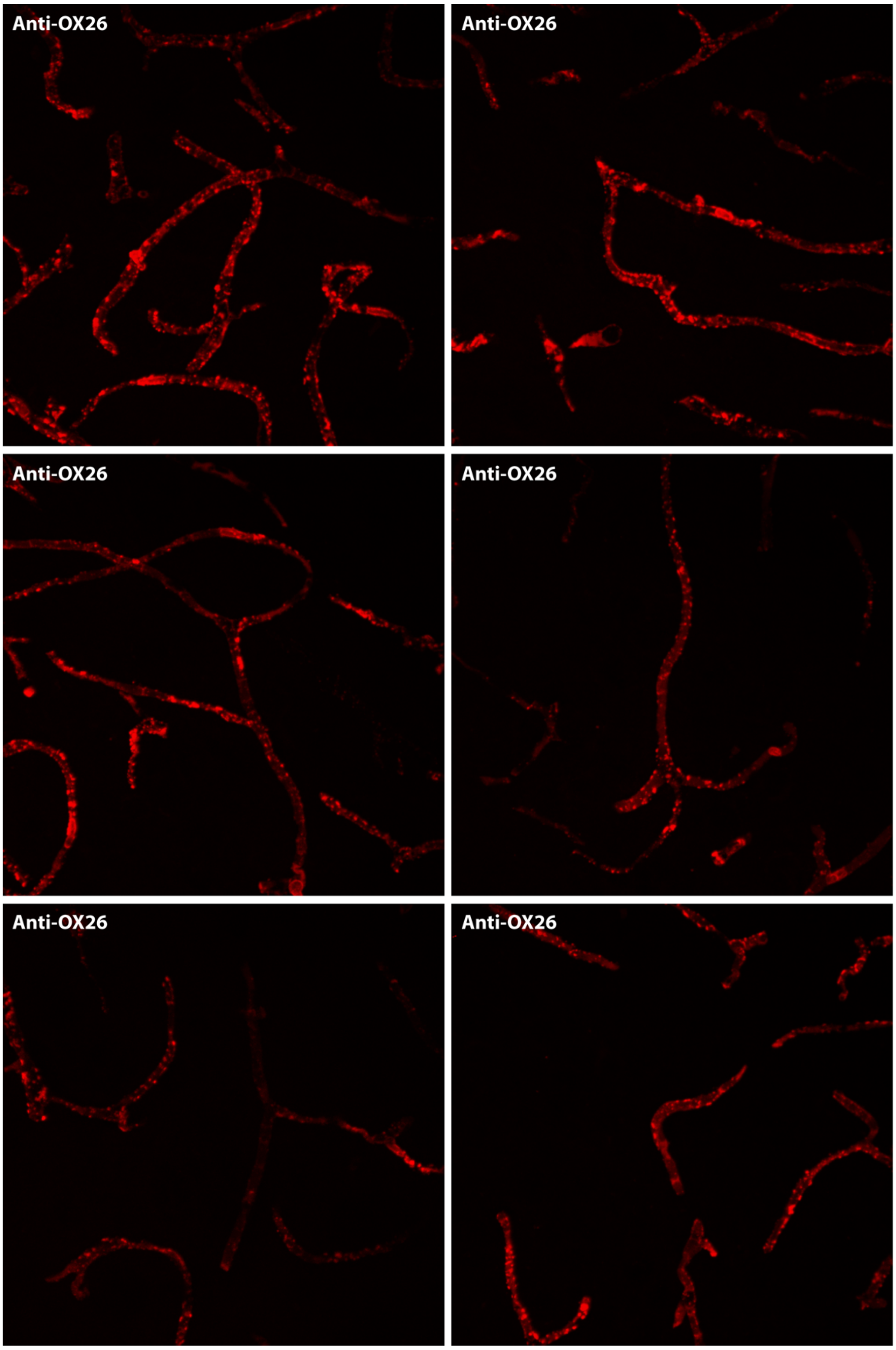

Materials and methods for *"Targeting transferrin receptors at the blood-brain barrier improves the uptake of immunoliposomes and subsequent cargo transport into the brain parenchyma"*

Kasper Bendix Johnsen[1,2], Annette Burkhart[1], Fredrik Melander[2], Paul Joseph Kempen[2], Jonas Bruun Vejlebo[2], Piotr Siupka[3], Morten Schallburg Nielsen[3], Thomas Lars Andresen[2] & Torben Moos[1]*

[1]Laboratory for Neurobiology, Biomedicine, Institute of Health Science and Technology, Aalborg University, Aalborg, Denmark. [2]Center for Nanomedicine and Theranostics, Department of Micro- and Nanotechnology, Technical University of Denmark, Denmark. [3]Department of Biomedicine, Aarhus University, Denmark.

Materials and methods

Materials

1,2-distearoyl-*sn*-glycero-3-phosphocholine (DSPC)(Cat. No. 850365), 1,2-distearoyl-*sn*-glycero-3-phosphoethanolamine-(polyethylene glycol)$_{2000}$ (DSPE-PEG$_{2000}$)(Cat. No. 8880120), 1,2-distearoyl-*sn*-glycero-3-phosphoethanolamine-N-[maleimide(polyethylene glycol)$_{2000}$] (DSPE-PEG$_{2000}$-maleimide)(Cat. No. 880126), 1-palmitoyl-2-(dipyrrometheneboron difluoride)undecanoyl-*sn*-glycero-3-phosphocholine (TopFluor PC)(Cat. No. 810281), and cholesterol (Cat. No. 700000) were purchased at Avanti Polar Lipids Inc. (Alabaster, AL, USA). A stealth liposome formulation lipid mixture of hydrogenated soybean phosphatidylcholine (HSPC), DSPE-PEG$_{2000}$ and cholesterol (56.8:38.2:5) was purchased at Lipoid GmbH (Ludwigshafen, Germany). Boric acid (Cat. No. B2645), *tert*-butanol (*tert*-BuOH)(Cat. No. 471712), hydrochloric acid (HCl)(Cat. No. 84415), nitric acid (HNO_3)(Cat. No. 84385), hydrogen peroxide solution (H_2O_2)(Cat. No. 95321), Percoll (Cat. No. P1644), collagen type IV (Cat. No. C5533), fibronectin (Cat. No. F1141), poly-L-lysine (Cat. No. P6282), heparin (Cat. No. H3149), sodium chloride (NaCl)(Cat. No. S7653), puromycin (Cat. No. P8833), 2-iminothiolane (Traut's reagent)(Cat. No. I6256), hydrocortisone (Cat. No. H4001), CTP-cAMP (Cat. No. C3912), 4-(3-butoxy-4-methoxybenzyl)imidazolidin-2-one (RO-201724)(Cat. No. B8279), 4-(2-Hydroxyethyl)piperazine-1-ethanesulfonic acid sodium salt (HEPES-Na)(Cat. No. H7006), Fluka platinum, gallium and iridium analytical standard solutions (Cat. No. 19078, 16639, 207209), insulin-transferrin-sodium selenite (Cat. No. 11074547001), 4,6-diamino-phenylindole dihydrochloride (DAPI)(Cat. No. D9542), and the OX26 hybridoma (Cat. No. 84112014) were purchased at Sigma-Aldrich (Brøndby, DK). DNase I (Cat. No. 1014159001), collagenase/dispase (Cat. No. 109113), and basic fibroblast growth factor (bFGF)(Cat. No. 1363697) were purchased at Roche (Hvidovre, DK). Rabbit anti-ZO-1 (Cat. No. 61-7300), Alexa Fluor 594-conjugated goat anti-mouse IgG (Cat. No. A11032), Alexa Fluor 594-conjugated goat anti-rabbit IgG (Cat. No. A11034), mouse IgG isotype control (Cat. No. 31903), collagenase II (Cat. No. 17101105), Dulbecco's Modified Eagle Medium (DMEM)(Cat. No. 21885), DMEM with F12 nutrient mixture (DMEM-F12)(Cat. No. 31331), and fetal calf serum (Cat. No. 10270) were purchased at Thermo Scientific (Hvidovre, DK). Bovine serum albumin (BSA)(Cat. No. EQBAH62) was purchased at Europa Bioproducts (Cambridge, UK). Plasma-derived bovine serum (Cat. No. 60-00-810) was purchased at First Link (Wolverhampton, UK). Gentamicin sulphate (G418)(Cat. No. 17-518Z) was purchased at Lonza Copenhagen (Vallensbæk Strand, DK). Fluorescence mounting medium (Cat. No. S3023) and

rabbit anti-glial acidic fibrillary protein (GFAP)(Cat. No. Z0334) were purchased at DAKO (Glostrup, DK). Hanging cell culture inserts (Transwell)(Cat. No. Pirp 15R48) were purchased at Merck Millipore (Hellerup, DK). Oxaliplatin was purchased at Lianyungang Guiyuan Chempharm Co., LTD (Jiangsu, China).

Thiolation of antibodies

OX26 (mouse anti-rat transferrin receptor antibody, produced in-house via the hybridoma technique) and isotype IgG control (mouse IgG, Thermo Fisher Scientific, Nærum, DK) antibodies were diluted to a final concentration of 2.5 mg/mL in 0.2 M sodium borate buffer (pH 8.5) mixed with 2-iminothiolane (Traut's reagent) at a molar ratio of 1:40. The molar ratio was chosen to obtain approximately 1.2 thiol groups per antibody as measured by Ellman's reaction (Supplementary Fig. S1). The thiolation reaction proceeded for 60 min at room temperature and the thiolated antibodies were then transferred to Amicon Ultra 4 mL spin filters (MW cut-off: 30 kDa). The spin filters were filled with 0.2 M sodium borate buffer and centrifuged at 2,000 rcf for 30 min at 4°C, and the filtrate discarded. This process was repeated two times to rinse the thiolated antibodies from unreacted 2-iminothiolane. The purified and thiolated antibodies were immediately mixed with DSPE-PEG$_{2000}$-maleimide micelles or DSPE-PEG$_{2000}$-maleimide liposomes.

Preparation of antibody micelles for post-insertion

DSPE-PEG$_{2000}$-methoxy and DSPE-PEG$_{2000}$-maleimide were solubilized in a *tert*-BuOH:water mixture (9:1), mixed in a 4:1 molar ratio in a glass vial, and lyophilized overnight. Thiolated antibody was added to the micelle powder in a molar ratio of 1:5 (as determined by the number of available maleimide groups), and the air phase of the glass vial was replaced with N_2 before incubation overnight on a rocking table (200 rpm) in the dark to allow for the antibodies to conjugate to the micelles. After incubation, the micelle-conjugated antibodies were purified from non-bound antibodies by size exclusion chromatography (SEC) using a Sepharose CL-4B column with a 10 mM HEPES, 150 mM NaCl (pH 7.4) buffer as the fluid phase. The SEC fractions were analyzed by the BCA assay (Thermo Scientific, Hvidovre, DK)(Supplementary Fig. S2), and the fractions containing micelle-conjugated antibodies up-concentrated using Amicon Ultra 4 mL spin filters (30 kDa MWCO, Merck Life Science, DK) at 2,500 rcf followed by extensive washing with MilliQ water. The protein concentration was measured in the final antibody-micelle preparation using the BCA assay, and the sample was lyophilized and stored at – 20°C.

Preparation of immunoliposomes

Fluorescently labelled immunoliposomes were prepared by the post-functionalization technique[1,2]. Lipids were solubilized in a *tert*-BuOH:water mixture (9:1) and mixed in the following ratio: DSPC/Cholesterol/DSPE-PEG$_{2000}$/DSPE-PEG$_{2000}$-maleimide/TopFluor PC (59.9/35/4.6/0.4/0.1) in glass vials and lyophilized overnight. The resulting lipid powder was hydrated in a 10 mM HEPES, 150 mM NaCl (pH 7.4) buffer for 60 min at 65°C to yield a final lipid concentration of approximately 20 mM. The multilamellar vesicles contained in the hydrated suspension were downsized to unilamellar liposomes by multiple extrusion through 100 nm polycarbonate filters using an Avanti Mini Extruder (Avanti Polar Lipids Inc., Alabaster, AL, USA). The liposome size and surface charge were estimated using dynamic light scattering and zeta potential measurement, respectively. For zeta-potential measurements, the liposomes were diluted in a 10 mM HEPES, 5 % (w/v) glucose buffer to avoid salt interference. The liposomes were then immediately mixed with thiolated antibodies in a molar ratio of 5:1 (as determined by available maleimide groups). The air phase of the glass vial was then replaced with N_2, and the liposome-antibody mixture was incubated overnight on a rocking table (200 rpm) in the dark to allow for conjugation. After the incubation, the antibody-functionalized liposomes were isolated from the non-bound antibodies by SEC using a Sepharose CL-4B column with a 10 mM HEPES, 150 mM NaCl (pH 7.4) buffer as the fluid phase. The SEC fractions were analyzed by the BCA assay (Thermo Scientific, Hvidovre, DK) for the antibody concentration and fluorescence intensity measurements for the liposome fluorescence emission (Supplementary Fig. S3). The fractions containing antibody-functionalized liposomes were up-concentrated using Amicon Ultra 4 mL spin filters (30 kDa MWCO, Merck Life Science, DK) at 1,500 rcf. The particle size and zeta potential were determined using a Zetasizer (ZetaPALS, Brookhaven Instruments Ltd., NY, USA)(Table 1). All liposome formulations were analyzed for their phosphor content by inductively-coupled plasma-mass spectrometry (ICP-MS, iCAP Q, Thermo Scientific)(see below) to determine the lipid concentration.

Oxaliplatin-loaded immunoliposomes were produced by the post-insertion technique. The dry lipid powder was hydrated to a concentration of 15 mg/mL in 10 mM HEPES and 5 % glucose (pH 7.4) for 1 hour at 65°C. The resulting liposomes were extruded with a high-pressure extruder (Northern Lipids Inc., Burnaby, Canada) by passing the liposomes two times through two stacked 200 nm polycarbonate filter (Whatman, Maidstone, UK) followed by five passes through two 100 nm filters. The temperature was maintained at 65°C during the extrusion process. The resulting liposomes

were mixed with antibody micelles in a ratio yielding approximately 50 antibodies per liposome (Table 1). Un-encapsulated free oxaliplatin was removed by dialysis using a dialysis cassette (Slide-A-Lyzer, 10 kDa MWCO, Pierce, Thermo Scientific, Hvidovre, DK) against 10 mM HEPES, 5 % glucose (pH 7.4). Encapsulated oxaliplatin and phospholipid content were measured by ICP-MS (see below). To measure the degree of encapsulation, the liposomes were subjected to spin filtration using Amicon Ultra spin filters (MW cut-off: 100 kDa, Merck Life Science, DK), and the oxaliplatin concentration measured in the filtrate. The particle size (as approximated using dynamic light scattering (DLS)) and zeta potential were determined with a Zetasizer (ZetaPALS, Brookhaven Instruments Ltd., NY, USA)(Table 1).

Across the nine batches of liposomes used for this study (both fluorescent and oxaliplatin-loaded), the mean hydrodynamic diameter was 145.7 ± 5.8 nm and 146.4 ± 6.3 nm for OX26 and isotype IgG immunoliposomes, respectively. The mean antibody functionalization was 54 ± 20 antibodies per liposome. For the oxaliplatin-loaded immunoliposomes used in the quantitative parts of the study, the mean hydrodynamic diameter was 139.3 ± 1.5 nm and 139.8 ± 2.0 nm for OX26 and isotype IgG immunoliposomes, respectively, whereas the mean antibody functionalization was 47 ± 9 for the OX26 immunoliposomes and 52 ± 7 for the isotype IgG immunoliposomes (Table 1).

Cryo-transmission electron microscopy

To evaluate the morphology of the liposomes, 3 μL liposome solution were placed on a lacy carbon 300 mesh copper transmission electron microscopy grid, blotted, and plunge frozen in liquid ethane using a FEI Vitrobot Mark IV. The samples were imaged using a FEI Tecnai G2 20 TWIN transmission electron microscope operated at 200 keV in low dose mode with a FEI High-Sensitive (HS) 4k x 4k Eagle camera. All imaging was done at – 175°C.

Cell culture

Primary cultures of rat brain capillary endothelial cells (BCECs) were prepared from two to three week old Sprague-Dawley rats using the protocol recently described in Burkhart et al. (2015)[3]. All experiments and handling of rats were approved by the Danish National Council for Animal Welfare (License no. 2013-15-2934-00893). The experiments were all performed in accordance with relevant guidelines and regulations (EU directive 2010/63/EU and relevant local additions to this directive).

The rats were anesthetized by isoflurane (Baxter, Søborg, DK), and their heads were rinsed in 70 % ethanol before decapitation. Brains were carefully dissected under sterile conditions, and the

forebrains were placed in ice-cold 0.1 M PBS (pH 7.4) until further processing. Meninges and white matter were removed from each hemisphere using filter paper and tweezers, and the resulting cortical tissue was cut into small pieces using sterile razor blades while preserved in ice-cold DMEM-F12. The tissue was then digested using collagenase II and DNase I in DMEM-F12 at 37°C for 75 min, after which the enzyme activity was inhibited by diluting the tissue-containing solution in additional DMEM-F12. After centrifugation at 1,000 rcf for 8 min, the pellet was resuspended in 20 % BSA in DMEM-F12, and centrifuged again at 1,000 rcf for 20 min. The microvessels contained in the pellet were treated with collagenase/dispase and DNase I in DMEM-F12 at 37°C for 50 min, and the resulting digested microvessels separated from other cell types on a continuous Percoll gradient (33 %). The microvessels were then collected from the Percoll gradient and seeded onto plastic dishes coated with collagen IV (0.15 mg/mL) and fibronectin (0.05 mg/mL). The primary BCECs were cultured in an incubator with 5 % CO_2 at 37°C in DMEM-F12 supplemented with 10 % plasma-derived bovine serum, bFGF, heparin, insulin-transferrin-sodium selenite and gentamicin sulphate. Puromycin (a p-gp substrate, 4 µg/mL) was added to the culture medium for the initial three days to remove any pericytes present in the BCEC isolate.

Primary astrocytes were isolated from neonatal Sprague-Dawley rats. The neonatal pups were decapitated, and their brains removed from the skull. The cerebral cortex was mechanically dissociated from the remaining brain using a 40 µm nylon cell strainer in DMEM supplemented with 10 % fetal calf serum and G418. Culture flasks were coated with poly-L-lysine (0.1 mg/mL), and dissociated cells seeded into these and allowed to grow to confluence. The astrocytes were then seeded on the bottom of 12-well plates for subsequent use in co-culture with BCECs in hanging inserts.

Construction of in vitro BBB models

Three days after isolation, the BCECs were detached from the culture dish and passaged onto hanging cell culture inserts fitted for 12-well plates (polyethylene terephthalate, pore size: 1 µm) and coated with collagen IV and fibronectin. After adherence overnight, the inserts were transferred into a new 12-well plate containing a confluent layer of astrocytes in each well. In addition to the stimulus provided by the presence of astrocytes, the BCECs were treated with hydrocortisone, cAMP and RO-201724 at concentrations of 550 nM, 250 µM and 17.5 µM, respectively, to further induce tightness in the resulting *in vitro* BBB model.

To determine the integrity of the *in vitro* BBB models derived from primary rat BCECs, the transendothelial electrical resistance (detecting the flux of sodium ions across the cell layer) was measured using the Millicell ERS-2 Epithelial Volt-Ohm meter and STX01 Chopstick Electrodes (Millipore, Hellerup Denmark, DK). All TEER measurement were normalized by subtracting the value derived from a coated insert without any cells and subsequently multiplied by the area of the culture insert (1.12 cm^2). The data was analysed in GraphPad Prism 5.0 (GraphPad Software, Inc., CA, USA), and the TEER values are presented as $\Omega*cm^2$.

Uptake studies evaluated by flow cytometry

To evaluate association and/or uptake of fluorescently labelled immunoliposomes into BCECs, *in vitro* BBB models were treated with either OX26 immunoliposomes, isotype IgG immunoliposomes or stealth liposomes (with no antibodies attached) (60 nmol lipid per well) and incubated for 2 hours. The cells were washed carefully three times using 0.1 mg/mL heparin in 0.1 M PBS (pH 7.4) and then detached from the culture insert membranes using trypsin. The trypsinized cells were transferred to flow cytometry tubes and centrifuged at 140 rcf for 5 min and resuspended in 0.1 M PBS (pH 7.4). The amount of association between immunoliposomes and BCECs were measured on a MoFlo® Flow Cytometer System™ (Beckman-Coulter, Copenhagen, DK). Prior to analysis, the flow cytometer parameters were calibrated using SPHERO™ Ultra Rainbow Fluorescent Particles (3 µm)(Spherotech, Lake Forrest, IL, USA). Cells were gated based on the forward/side scatter plot to eliminate cell debris in the subsequent analysis. The fluorescence intensity of the immunoliposome-treated cells was corrected based on gating of the autofluorescence of non-treated cells. The resulting data was analyzed using Kaluza software (Beckman-Coulter, Copenhagen, DK), and the median fluorescence intensity of the individual treatment groups was plotted in GraphPad Prism 5.0 (GraphPad Software, Inc., CA, USA).

Each preparation of fluorescently labelled immunoliposomes were analyzed for their binding capacity to the immortalized rat brain endothelial cell line, RBE4, prior to any experiments using primary rat brain endothelial cells. The cells were seeded in collagen I-coated 12-well plates and grown to a monolayer in Alpha-MEM + Ham's F10 (Thermo Scientific, Hvidovre, DK) with 10 % FCS, 300 µg/mL G418 (Lonza Copenhagen, Vallensbæk Strand, DK) and 1 ng/mL bFGF (Roche, Hvidovre, DK) before administration of fluorescently labelled immunoliposomes in the concentration described above. After incubation, the cells were washed and trypsinized as described above, and analyzed by flow cytometry using a Gallios™ Flow Cytometer (Beckman-Coulter, Copenhagen,

DK). To investigate the possible cross-reactivity of the prepared immunoliposomes to the mouse transferrin receptor, the immortalized mouse brain endothelial cell line, bEnd.3, was included in experiments like those above (Supplementary Fig. S4). The bEnd.3 cells were cultured in DMEM containing 10 % FCS and 1 % penicillin and streptomycin.

Uptake and transcytosis studies using oxaliplatin-encapsulated immunoliposomes

After reaching high TEER (regarded as > 150 Ω*cm^2 as suggested by Burkhart et al. (2015)[3]), the BCECs were treated with oxaliplatin-loaded immunoliposomes (OX26 or isotype IgG) at a lipid concentration of 120 µM corresponding to total amount of 2.65 µg oxaliplatin per insert. Immediately after the administration, a sample was taken from each insert to measure the exact platinum content administered. The BCECs were incubated for 4 hours to allow for liposome/oxaliplatin uptake and transport across the membrane. The TEER was measured again after the incubation to ensure the barrier integrity was maintained during the experiment (Supplementary Fig. S5). Afterwards, samples were taken from the culture medium in the insert ('blood fraction') and the bottom well ('brain fraction', see Fig. 4A). The BCECs in the insert were carefully washed three times using 0.1 mg/mL heparin in 0.1 M PBS (pH 7.4) and detached from the culture insert membranes using trypsin ('BCEC fraction'). The platinum content in each sample was measured using ICP-MS (see below), and the resulting data was analysed in GraphPad Prism 5.0 (GraphPad Software, Inc., CA, USA). The results for platinum uptake and transport was plotted as percentage of administrated dose (%AD) as depicted from the sample taken immediately after administration.

Immunocytochemistry and lysosome staining

To evaluate the intracellular location of the immunoliposomes after endocytosis into BCECs, immunocytochemistry was performed. Prior to staining with EEA-1 antibodies or LysoTracker, the BCECs were treated with either OX26 or isotype IgG immunoliposomes (60 nmol lipid per well) and incubated for 2 hours. BCECs in the culture inserts were washed in 0.1 M PBS (pH 7.4) and fixed in 4 % paraformaldehyde in 0.1 M PBS (pH 7.4) for 15 min. After three washes in 0.1 M PBS (pH 7.4), the BCECs were permeabilized and unspecific binding blocked with 0.05 % saponin and 3 % BSA in 0.1 M PBS (pH 7.4) for 30 min. BCECs were then incubated with primary antibodies against ZO-1, transferrin receptor and EEA-1 that marks early endosomes (all primary antibodies were diluted 1:200 in 1 % BSA in 0.1 M PBS (pH 7.4)) and incubated for 1 hour at room temperature. Alexa Flour 594-conjugated goat-anti-rabbit or Alexa Flour 594-conjugated goat-anti-mouse secondary

antibodies were added in a dilution of 1:200 in washing buffer (1:50 dilution of incubation buffer) and incubated for 1 hour at room temperature. The nuclei were then stained with diamino-phenylindole (DAPI), the culture insert membranes were mounted on glass slides with fluorescence mounting medium (DAKO, Glostrup, DK), and confocal imaging was performed using an Olympus IX-83 fluorescent microscope with an Andor confocal spinning disk unit and an Andor iXon Ultra 897 camera, using Olympus cellSens software. Images of cells were taken using a UPLSAPO 100X magnification objective lens (NA 1.40). All images were processed using Fiji software[4].

For lysosomal staining, the BCECs were treated with LysoTracker Red DND-99 (Thermo Scientific, Hvidovre, DK) following the manufacturer's protocol, and the cells were incubated for 30 min. The BCECs in the culture inserts were washed in 0.1 M PBS (pH 7.4) and fixed in 4 % paraformaldehyde in 0.1 M PBS (pH 7.4) for 15 min. The nuclei were stained with DAPI.

In vivo studies and tissue isolation

All procedures and handling of rats were approved by the Danish National Council for Animal Welfare (License no. 2013-15-2934-00893). The experiments were all performed in accordance with relevant guidelines and regulations (EU directive 2010/63/EU and relevant local additions to this directive). Uptake studies *in vivo* were performed using male P18-P20 Sprague-Dawley rats (n = 5 per group), which were intravenously injected through the lateral tail vein with fluorescently labelled immunoliposomes for morphological studies, or oxaliplatin-loaded immunoliposomes or free oxaliplatin for brain uptake and biodistribution studies.

For morphological analysis, fluorescently labelled immunoliposomes were injected (5 µmol lipid per animal) and allowed to circulate for 2 hours. Afterwards, the rats were deeply anesthetized by a subcutaneous injection of 0.5 ml/10 g body weight of Hypnorm/Dormicum (fentanyl/fluanisone mixed with midazolam and sterile water in a ratio of 1:1:2). The chest cage was opened, and the rats transcardially perfused with 0.1 M PBS (pH 7.4) followed by 4 % paraformaldehyde in 0.1 M PBS (pH 7.4). The fixed rats were decapitated and the skull opened to expose the brain. The brains were submerged into fixative for 24 hours followed by extensive washing in KPBS and immersion in 30 % sucrose. Brains were then sectioned into 40 µm slices and kept at - 20°C in anti-freeze solution until further processing.

For quantitative brain uptake and biodistribution studies, the oxaliplatin-loaded immunoliposomes (5 µmol lipid per animal corresponding to 186 µg oxaliplatin) or free oxaliplatin (186 µg per animal) were injected, and allowed to circulate for 0, 0.5, 1, 4, and 24 hours. After the

circulation, the rats were deeply anesthetized by a subcutaneous injection of 0.5 ml/10 g body weight of Hypnorm/Dormicum (fentanyl/fluanisone mixed with midazolam and sterile water in a ratio of 1:1:2). The chest cage was opened, and the rats were transcardially perfused with 0.1 M PBS (pH 7.4). After perfusion, the skull was opened and the brains removed. One hemisphere of the brain was quickly frozen on dry ice, and the other used for brain capillary depletion (see below). Tissue samples were also taken from the liver, spleen, kidney, lung and heart, which were quickly frozen on dry ice too and stored until processing for ICP-MS.

Before perfusion of the animals with 0.1 M PBS (pH 7.4), blood samples were drawn from the left ventricle of the heart into a heparin-containing tube (time points: 0, 0.5, 1, 4, and 24 hours). The blood samples were centrifuged at 2,000 rcf for 15 min to pellet cells and platelets, and the plasma transferred into clean tubes and stored at – 20°C. The platinum content was measured using ICP-MS (see below) and the resulting data plotted as %ID/g in GraphPad Prism 5.0 (GraphPad Software, Inc., CA, USA).

Immunohistochemistry

Brain sections from the fluorescently-labelled liposome-injected animals were washed three times in KPBS, and blocked and permeabilized with 3 % swine serum and 0.1 % Triton X-100 in KPBS (blocking buffer) for 30 min at room temperature. Since the primary antibodies in this experiment was the ones conjugated to the liposome surface, only Alexa Flour 594-conjugated goat anti-mouse IgG was added to the brain sections at a dilution of 1:200 in blocking buffer and incubated on a rocking table for 60 min at room temperature. After incubation, the sections were washed three times in washing buffer (1:50 dilution of blocking buffer in KPBS), and mounted on glass slides. Confocal imaging was performed using an Olympus IX-83 fluorescent microscope with an Andor confocal spinning disk unit and an Andor iXon Ultra 897 camera, using Olympus cellSens software. Images of cells were taken using a UPLSAPO 60X magnification objective lens (NA 1.20). All images were processed using Fiji software[4].

Brain capillary depletion

The presence of transcytosed platinum in the brain parenchyma was evaluated by separating the brain capillaries using the brain capillary depletion technique of Triguero et al. (1990) with minor modifications[5]. One hemisphere per rat was homogenized by four strokes in a dounce homogenizer in 3.5 mL ice-cold homogenization buffer (10 mM HEPES, 141 mM NaCl, 4 mM KCl, 2.8 mM

$CaCl_2$, 1 mM $MgSO_4$, 1 mM NaH_2PO_4, and 10 mM glucose; pH 7.4). The homogenized brain tissue was mixed with 3.5 mL ice-cold 30 % dextran (MW: 60,000, Sigma-Aldrich), and the mixture was further homogenized by four strokes. The homogenate was then transferred to a 15 mL Falcon tube and centrifuged at 3,500 rcf for 40 min at 4°C with slow deceleration. Afterwards, the supernatant was removed and stored separately from the capillary-containing pellet. Samples were taken from both fractions and processed for ICP-MS to quantify the amount of platinum (see below). The purity of the depleted capillaries was determined by the expression of alkaline phosphatase, and corresponded to what was previously shown in the rat[6,7].

Inductively-coupled plasma mass spectrometry

To analyse the platinum content in the extracted tissue samples, a maximum of 100 mg tissue was digested in aqua regia overnight at 65°C. After complete digestion, the samples were diluted in MilliQ water containing 0.5 ppb iridium (Fluka, Sigma-Aldrich, Brøndby, DK) followed by dilution in 2 % HCl containing 0.5 ppb iridium. All samples were analyzed on a iCAP Q ICP-MS system (Thermo Scientific, Hvidovre, DK) fitted with an ASX-520 AutoSampler and a Neclar ThermoFlex 2500 chiller. Prior to analysis, the instrument was calibrated using TUNE B iCAP Q element mixture (Thermo Scientific, Hvidovre, DK), and a standard curve was generated based on serial dilution of an analytical standard platinum solution (Fluka, Sigma-Aldrich, Brøndby, DK) to obtain data points ranging from 0.08 – 10 ppb. In addition to analysing the platinum content in each sample, the iridium content was measured as an internal standard. The resulting data was plotted as %ID/g in GraphPad Prism 5.0 (GraphPad Software, Inc., CA, USA).

To measure the content of phosphor in the liposome formulations (as an indicator of phospholipid concentration), a sample of liposomes was diluted in 2 % HCl containing 0.5 ppb gallium (Fluka, Sigma-Aldrich, Brøndby, DK). All samples were analysed as described above with a standard curve generated from serial dilution of an analytical standard phosphor solution (Fluka, Sigma-Aldrich, Brøndby, DK).

References

1. Jølck, R. I., Feldborg, L. N., Andersen, S., Moghimi, S. M. & Andresen, T. L. Engineering liposomes and nanoparticles for biological targeting. *Adv. Biochem. Eng. Biotechnol.* **125,** 251–280 (2011).
2. Bak, M., Jølck, R. I., Eliasen, R. & Andresen, T. L. Affinity Induced Surface Functionalization of Liposomes Using Cu-Free Click Chemistry. *Bioconjug. Chem.* **27,** 1673–1680 (2016).
3. Burkhart, A. *et al.* Transfection of brain capillary endothelial cells in primary culture with

defined blood-brain barrier properties. *Fluids Barriers CNS* **12,** 19 (2015).

4. Schindelin, J. *et al.* Fiji: an open-source platform for biological-image analysis. *Nat. Methods* **9,** 676–682 (2012).
5. Triguero, D., Buciak, J. & Pardridge, W. M. Capillary depletion method for quantification of blood-brain barrier transport of circulating peptides and plasma proteins. *J. Neurochem.* **54,** 1882–1888 (1990).
6. Moos, T. & Morgan, E. H. Restricted transport of anti-transferrin receptor antibody (OX26) through the blood-brain barrier in the rat. *J. Neurochem.* **79,** 119–129 (2001).
7. Gosk, S., Vermehren, C., Storm, G. & Moos, T. Targeting anti-transferrin receptor antibody (OX26) and OX26-conjugated liposomes to brain capillary endothelial cells using in situ perfusion. *J. Cereb. Blood Flow Metab.* **24,** 1193–1204 (2004).
